# The suitability of micronuclei as markers of relative biological effect

**DOI:** 10.1093/mutage/geac001

**Published:** 2022-02-08

**Authors:** Charlotte J Heaven, Hannah C Wanstall, Nicholas T Henthorn, John-William Warmenhoven, Samuel P Ingram, Amy L Chadwick, Elham Santina, Jamie Honeychurch, Christine K Schmidt, Karen J Kirkby, Norman F Kirkby, Neil G Burnet, Michael J Merchant

**Affiliations:** 1 Division of Cancer Sciences, Faculty of Biology, Medicine and Health, School of Medical Sciences, The University of Manchester, Oxford Road, M13 9PL Manchester, United Kingdom; 2 Manchester Academic Health Science Centre, The Christie NHS Foundation Trust, Wilmslow Road, M20 4BX Manchester, United Kingdom; 3 Department of Physics and Astronomy, Faculty of Science and Engineering, The University of Manchester, Oxford Road, M13 9PL Manchester, United Kingdom; 4 Christie Medical Physics and Engineering, The Christie NHS Foundation Trust, Wilmslow Road, M20 4BX Manchester, United Kingdom

**Keywords:** micronuclei, cytochalasin-B, radiotherapy, DNA damage

## Abstract

Micronucleus (MN) formation is routinely used as a biodosimeter for radiation exposures and has historically been used as a measure of DNA damage in cells. Strongly correlating with dose, MN are also suggested to indicate radiation quality, differentiating between particle and photon irradiation. The “gold standard” for measuring MN formation is Fenech’s cytokinesis-block micronucleus (CBMN) cytome assay, which uses the cytokinesis blocking agent cytochalasin-B. Here, we present a comprehensive analysis of the literature investigating MN induction trends *in vitro*, collating 193 publications, with 2476 data points. Data were collected from original studies that used the CBMN assay to quantify MN in response to ionizing radiation *in vitro*. Overall, the meta-analysis showed that individual studies mostly have a linear increase of MN with dose [85% of MN per cell (MNPC) datasets and 89% of percentage containing MN (PCMN) datasets had an *R*^2^ greater than 0.90]. However, there is high variation between studies, resulting in a low *R*^2^ when data are combined (0.47 for MNPC datasets and 0.60 for PCMN datasets). Particle type, species, cell type, and cytochalasin-B concentration were suggested to influence MN frequency. However, variation in the data meant that the effects could not be strongly correlated with the experimental parameters investigated. There is less variation between studies when comparing the PCMN rather than the number of MNPC. Deviation from CBMN protocol specified timings did not have a large effect on MN induction. However, further analysis showed less variation between studies following Fenech’s protocol closely, which provided more reliable results. By limiting the cell type and species as well as only selecting studies following the Fenech protocol, *R*^2^ was increased to 0.64 for both measures. We therefore determine that due to variation between studies, MN are currently a poor predictor of radiation-induced DNA damage and make recommendations for futures studies assessing MN to improve consistency between datasets.

## Introduction

Micronuclei (MN) are a common biological marker used to measure DNA damage from radiation and chemical agents [1]. They are small nuclear bodies containing DNA that has been segregated from the main nucleus [2]. MN form from acentric fragments left unrepaired following DNA damage, or from lagging chromosomes during mitosis. The nuclear envelopes of MN are fragile in comparison with the main nucleus, resulting in an increased likelihood of rupture and release of DNA into the cytosol [3, 4]. DNA damage, and acentric fragments, are known to increase with radiation dose [5, 6]. Increased radiation doses, therefore, also increase the frequency of MN, meaning that MN have been used as a biomarker for radiation exposure [7].

MN are a popular method for measuring DNA damage due to the relatively uncomplicated techniques required to visualize them. The invention of the cytokinesis-block micronucleus (CBMN) cytome assay in 1985 by Fenech and Morley improved the accuracy of MN quantification, and has become the gold standard for MN scoring over the years [8].The assay uses cytochalasin-B (cyt-B) to inhibit spindle assembly and prevent cytokinesis following DNA replication [9, 10]. The result is that asynchronous cells are halted at the second cytokinesis following radiation treatment, ensuring MN produced or lost in consequent cell divisions are not included. In the original CBMN protocol [8] (published in full in 2007 [11]), cyt-B is added 44 h after irradiation and incubated for 24–28 h before cells are processed and analyzed. The protocol was designed for human lymphocytes, cultured from whole blood, and suggests using a cyt-B concentration of between 3 and 6 µg/ml, with the authors stating that concentration of cyt-B should be optimized for cell type.

Interest in MN has once again peaked due to their association with immune responses through the cyclic GMP–AMP synthase/stimulator of interferon genes (cGAS/STING) pathway [12]. The cGAS/STING pathway responds to cytosolic DNA or RNA usually produced from viral or bacterial infection and triggers a type 1 interferon led immune reaction. However more recently, DNA damage has also been shown to activate the pathway through the production of cytosolic DNA, leading to interest in radiation-induced immune responses [13]. Although there is still significant interest in mitochondrial DNA triggered STING, it is suggested that the main cause of cGAS/STING pathway upregulation following irradiation is DNA released following MN envelope rupture [14]. The rupture of a MN envelope releases DNA into the cytosol which is detected by cGAS [15]. This results in the release of the small molecule cyclic guanosine monophosphate–adenosine monophosphate which triggers STING. The activation of STING results in its dimerization and translocation from the endoplasmic reticulum toward the Golgi apparatus and the phosphorylation of IRF-3 [16]. This ultimately leads to the production of type I interferons and other downstream proinflammatory cytokines [17]. Given the interest in cGAS/STING stimulation, understanding of how and when MN form and their subsequent envelope rupture has become paramount. The reliability with which different radiation doses and modalities result in MN production is key in studying the triggers for this pathway and in future planning of concurrent radioimmunotherapy scheduling.

Although MN are a common biomarker for DNA damage, the reliability and accuracy of the CBMN assay has not yet been evaluated. This work presents a comprehensive meta-analysis of MN trends in response to radiation dose *in vitro*, alongside evaluating any effect from physical or biological factors. Also studied were any effects of experimental protocol including cyt-B concentration and CBMN protocol timings.

## Methodology

### Literature search strategy

The studies contained in this review were initially identified through a SCOPUS database search including the terms “∗radiation” AND “micronuclei”. Papers collected were published from 1985 when the CBMN assay was first published by Fenech and Morley [8], up to October 2020 when the literature search commenced. The resulting publications were subsequently assessed for suitability by the abstract. Final inclusion of the identified studies was determined by a full-text review according to the criteria specified in Inclusion and exclusion criteria.

### Inclusion and exclusion criteria

The following criteria identified eligible studies: MN studied in directly irradiated cells and raw data printed in table or graphical format, *in vitro* irradiation, CBMN assay used, published in English, clinically relevant dose (≥1 cGy) and dose rates (≤10 Gy/min) used. Exclusion criteria included: studies where an unirradiated control was not published, studies where dose or MN number could not be calculated from information in the methodology, and studies where cells were not representative of a normal asynchronous population at time of irradiation.

### Data extraction

The following information was extracted from each study: authors, title, publication year, physical dose, micronuclei induction, any error bars published and type of error, irradiation type, photon/ particle energy, linear energy transfer (LET), originating cell species, cell type/ cell line if used, cyt-B concentration, time between irradiation and cyt-B addition, time between cyt-B addition and cell fixation.

Where LET was not reported in the study, the LET was estimated based on information present in the paper. Here, the LET estimation was based on calculation using Geant4-DNA simulation [18–20]. The reported particle type and energy were simulated across a 10-µm water volume and the final LET was taken as the average energy loss per micron for primary particles only. Data on MN frequency were collected from tables published within the papers or extracted from graphical data. MN frequency data were divided into two categories depending on how it was measured: “MN per cell” (abbreviated to MNPC for this report), where the total number of MN had been counted and divided by the total number of binucleated cells scored; or “percentage of cells containing MN (%)” (abbreviated to PCMN) where binucleated cells had been scored as either containing at least one MN or not containing any. In some studies that supplied information about MNPC, it was possible to also calculate a value for PCMN provided data on zero MNPC was scored. When data from several volunteers were reported separately, the average of all volunteers was used, provided each volunteer was reported to be healthy and the same methodology was used for each sample. Where methodology was not explicity stated in the paper but referenced, details of cyt-B concentration and timings were extracted from the referenced paper.

### Statistical analysis

All statistical analyses were performed using R (v3.6.1) [21] and figures were produced using the package ggplot2 [22]. Where applicable, control values were removed from all points to remove natural variation. Statistical significance of dose on MN was assessed using a two-sample *t*-test, and variable effects on dose and MN were analyzed using two-way ANOVAs. A *P* value <.05 was considered statistically significant.

## Results and analysis

### Identification and characteristics of studies

**Figure 1.**
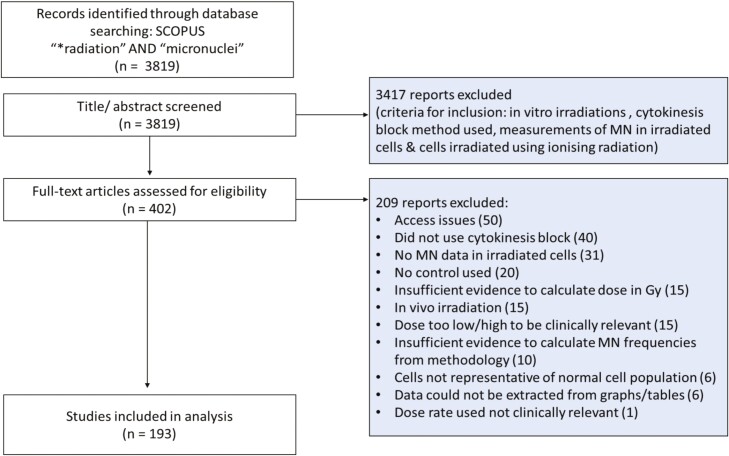
PRISMA flow showing how the studies included in the analysis were selected and reasons for the exclusion of other studies. Some studies passed initial phases of the screening due to lack of detail in the title/abstract, however these were later excluded following full-text review. For this reason, some exclusion criteria included are listed twice.

The PRISMA flow diagram for study selection is detailed in Fig. 1. In total, 193 studies were identified and included in the analysis which resulted in 2476 data points.

**Figure 2.**
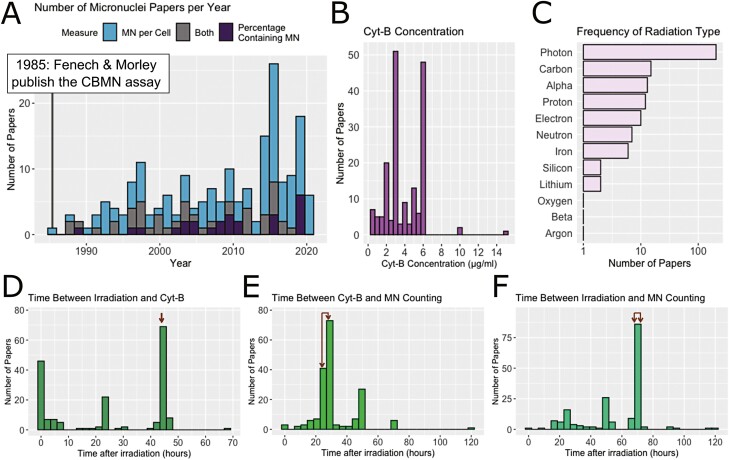
Characteristics of studies included in this meta-analysis. (A) The number of studies using the CBMN assay since its conception in 1985. (B) Cyt-B concentration used (some studies used more than one concentration). (C) Frequency of radiation type used in different studies. (D) Time between irradiation and cyt-B addition. (E) Time between cyt-B addition and MN fixation. (F) Overall time between irradiation and fixation. Red arrows indicate the timings detailed by the Fenech protocol (44, 24–28, and 68–82 h for D, E, and F, respectively).

The characteristics of the included studies are detailed in Fig. 2. Both methodologies for counting micronuclei (MNPC and PCMN, see methods for definitions) have been used over the inclusion period, with MNPC being the most common method (Fig. 2A). Photon irradiation was the most common irradiation type (96.9% of studies). Particle irradiation was less frequent, with the most common particle types being carbon, alpha, proton, and electrons (Fig. 2B). The experimental protocol varied between studies, with a variety of cyt-B concentrations (Fig. 2C) and time points (Fig. 2D–F) reported. The protocol timings suggested in the Fenech protocol are indicated using red arrows.

### Effect of dose on micronuclei induction

**Figure 3.**
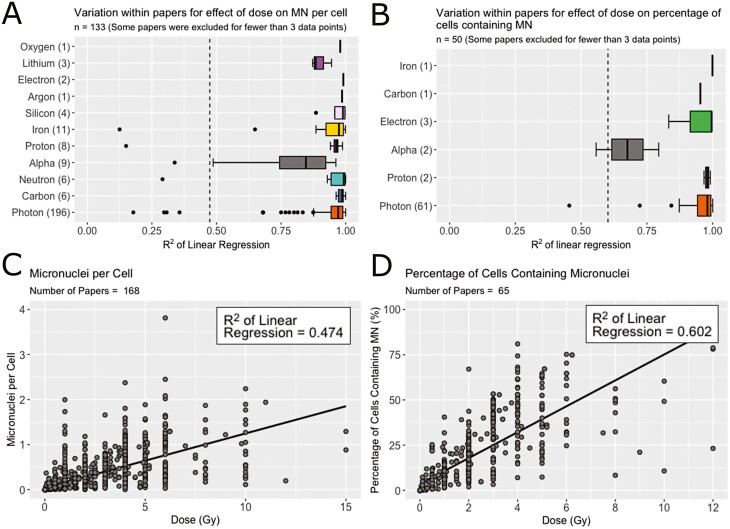
Effect of dose on MN. *R*^2^ of linear regression between (A) MNPC and (B) PCMN and dose split by radiation type. Papers with fewer than three data points were excluded as a linear regression could not be performed. Some papers contained more than one dataset; in this case separate regressions were analyzed. (C) Combined data for MNPC as a function of dose with a linear regression line for dose (*R*^2^ = 0.474). (D) Combined data for PCMN as a function of dose (*R*^2^ = 0.602). The *R*^2^ for the whole datasets (C or D) is shown in a dotted line on graphs A and B.

All studies included in the analysis reported an effect of dose on MN, which can usually be captured by a linear fit. Before combining the datasets, the strength of the correlation between MN and dose was quantified using the *R*^2^ of a linear regression within each paper. Where studies presented several radiation or cell types, separate regressions were conducted for each dataset. The results for the *R*^2^ values generated are presented in Fig. 3A for MNPC and Fig. 3B for PCMN, where they are split by radiation type. Overall, 85% of MNPC datasets and 89% of PCMN datasets had an *R*^2^ greater than 0.90, meaning that 90% of the variation in MN can be explained by dose.

All datasets were combined for MNPC and PCMN (Fig. 3C and D, respectively). The *R*^2^ of the linear regression between dose and MN was calculated to be 0.474 for MNPC and 0.602 for PCMN. Despite this large variation, in both cases, the effect of dose on MN was found to be significant (*P* < .001).

### Effect of physical and biological factors on micronuclei induction

**Figure 4.**
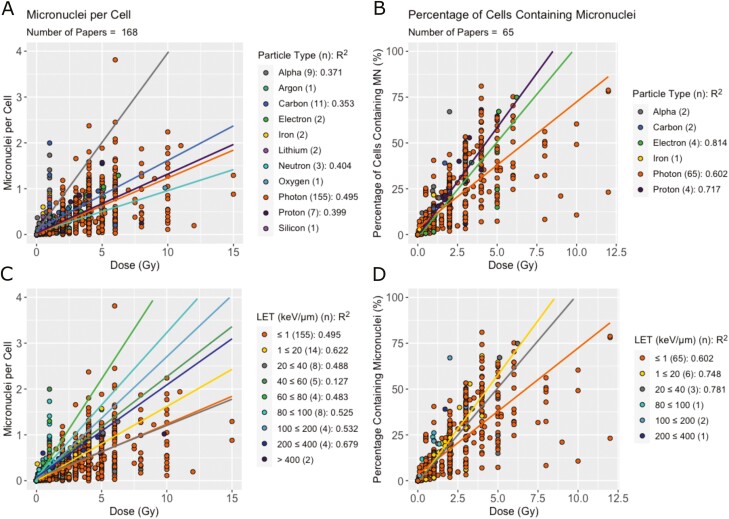
Effect of physical factors on MN produced per dose. (A) MNPC per dose and (B) PCMN per dose split by particle type. The number of studies presenting results for each particle type is shown in brackets. Linear regressions were performed where there were three or more papers and are shown with trend lines and *R*^2^ values on the figure. (C) MNPC and (D) PCMN per dose split by LET.

The MNPC and PCMN datasets were split by physical factors, particle type (Fig. 4A and B) and LET (Fig. 4C and D). Particle type was found to be a significant contributor to MN produced per dose in both cases (*P* < .001) and LET was found to be significant in the PCMN (*P* < .001) but not in MNPC. In both measures, photons produced fewer MN than protons for the same dose. In MNPC, studies reporting data from alpha particles had particularly high numbers of MN. Despite this, the variance within each dataset still resulted in poor *R*^2^ values, making it difficult to distinguish between physical variables.

**Figure 5.**
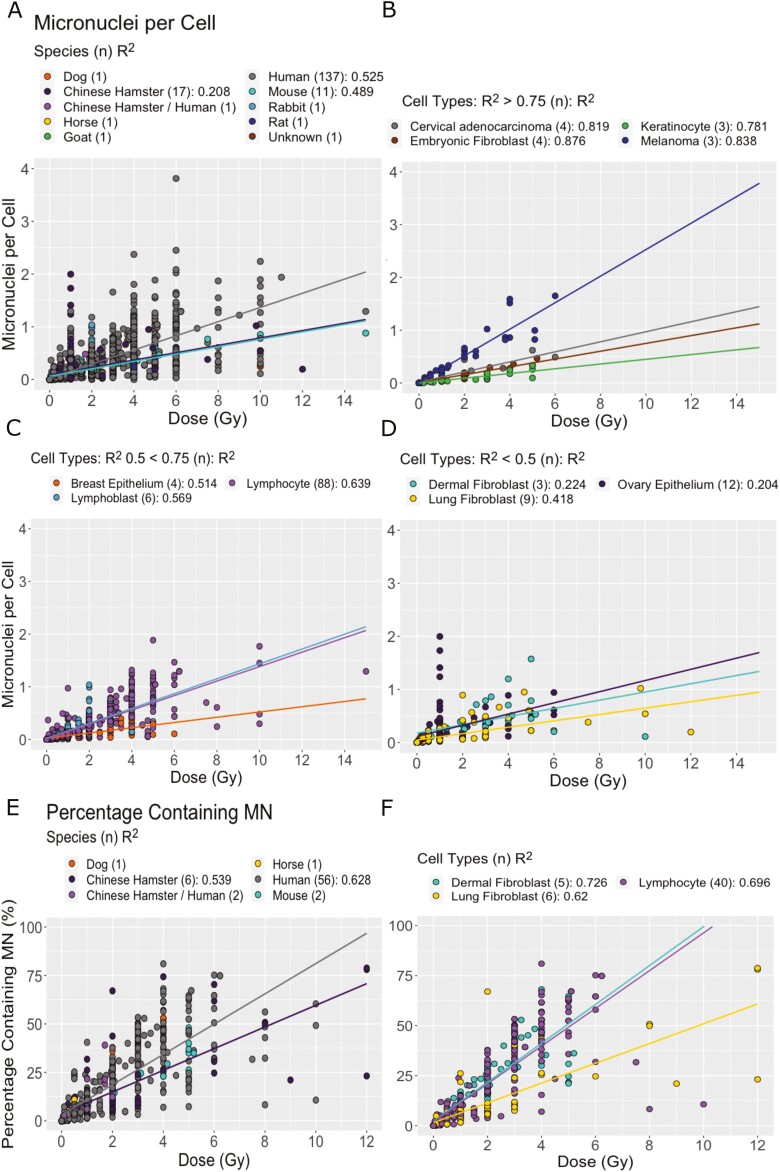
Effect of species and cell type on MN produced per dose. (A) MNPC split by species. (B–D) MNPC split by cell type where there are more than three papers. Cell types with (B) *R*^2^ > 0.75, (C) *R*^2^ between 0.5 and 0.75, and (D) *R*^2^ < 0.5. (E) PCMN split by species. (F) PCMN split by cell type where there are three or more papers. For all graphs, the number of studies is shown in brackets in the legend and *R*^2^ is calculated from linear regression where there are three or more studies included.

Species was also found to have an effect of MN produced per dose (Fig. 5A and E, *P* < .001). Both measures suggest that Chinese hamster cells are less sensitive to MN induction than human cells. MNPC also suggested this may be true of mouse cells. The *R*^2^ within species remained poor suggesting this may be contributing toward variation but was not responsible for all the variation.

There was a significant effect of different cell types on both MN measures (*P* < .001). The *R*^2^ of each cell type varied, however, some cell types being more consistent across studies than others (Fig. 5B–D and F). Melanoma cells appeared to have the highest MNPC per dose and keratinocytes the lowest. However, breast epithelium cells also appeared to produce few MN and ovary epithelial cells had very little correlation between MN produced and dose.

**Figure 6.**
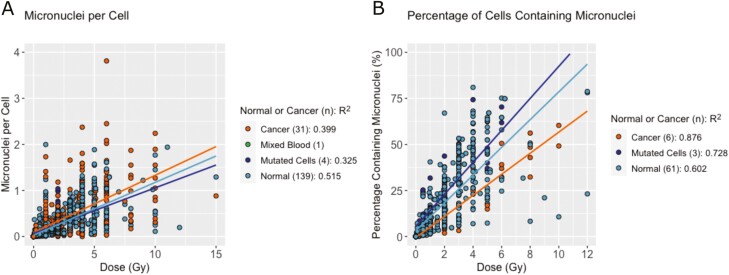
Rate of MN induction for cancer vs normal cells per dose. (A) MNPC and (B) PCMN per dose split by normal or cancer cell type. Mutated cells were separated as cells which had possible cancer mutations but had not been proven to be cancerous. One study averaged the healthy and cancer patient results, this has been separated from the other points and is referred to as “mixed blood.” The number of studies is shown in brackets in the legend and *R*^2^ is calculated from linear regression where there are three or more studies included.

No differences could be established in MNPC when considering normal vs cancer cells; however cancer cells produced a significantly lower PCMN per dose (*P* < .01, Fig. 6A and B).

### Effect of experimental factors on micronuclei induction

**Figure 7.**
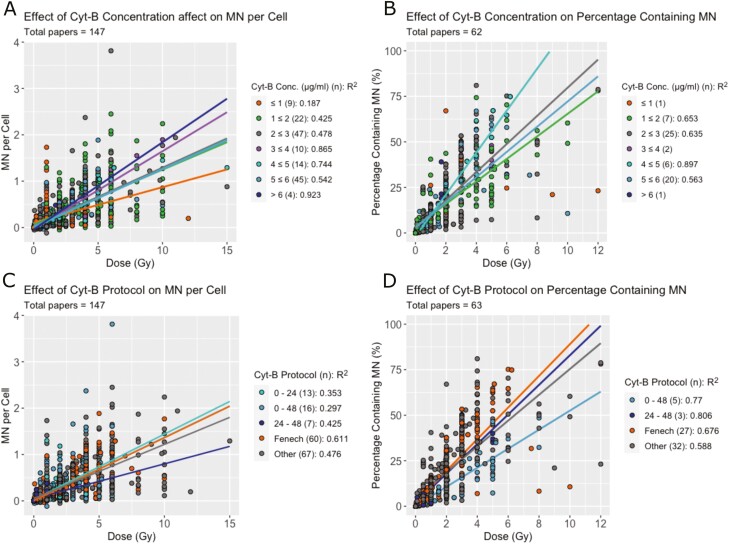
Experimental protocol effects on MN produced per dose. (A) MNPC per dose and (B) PCMN per dose split by cyt-B concentration. The number of studies presenting results for each concentration is shown in brackets. Linear regressions were performed where there were three or more studies and are shown with trend lines and *R*^2^ values on the figure. (C) MNPC per dose and (D) PCMN per dose split by common protocol timings including the Fenech protocol (orange) [11].

Experimental factors were also considered when analyzing variation in the datasets. Cyt-B concentration was found to be significant in both measures of MN (*P* < .001, Fig. 7A and B); however, this only had effects when in extremes (<1 or >6 µg/ml). Protocol timings had a significant effect on MNPC and PCMN (*P* < .05 and *P* < .001, respectively); however, the papers which more closely followed the Fenech protocol timings had less variation in their data. The *R*^2^ value for the Fenech protocol in MNPC was increased from 0.474 to 0.611 and for PCMN, the *R*^2^ increased from 0.602 to 0.676 (Fig. 7C and D). Other common timings used by several papers did not have the same effect on both measures.

### Subpopulation of micronuclei studies

**Figure 8.**
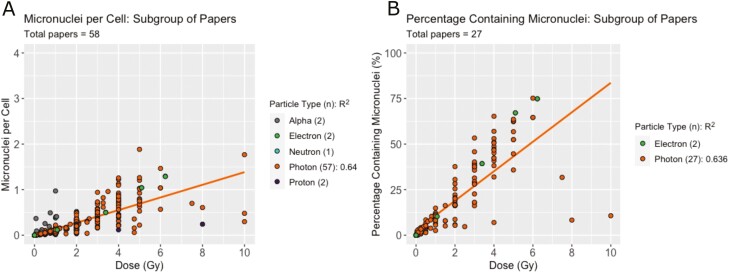
Subpopulation of studies effect of dose and particle type on MN. (A) MNPC and (B) PCMN produced at each dose split by particle type for papers only studying human lymphocytes and following the Fenech protocol timings. The number of studies presenting results for each particle type is shown in brackets. Linear regressions were performed where there were three or more studies and are shown with trend lines and *R*^2^ values on the figure.

A subpopulation of studies was chosen based on the above variables. Human lymphocytes were the most studied cell and were therefore selected to control for cell type and species. The protocol timings were also controlled for and only papers that followed Fenech’s protocol were used. The resulting 58 papers for MNPC and 27 papers for PCMN are shown in Fig. 8A and B, split by particle type. A marked increase can be seen in the *R*^2^ for the photon data when compared with the entire set of studies, with *R*^2^ increasing from 0.495 to 0.640 for MNPC and from 0.597 to 0.636 for PCMN. Two datasets studying the PCMN in this subpopulation have a large contribution away from the mean. Excluding these, the remaining 25 papers have an *R*^2^ of 0.89, suggesting 89% of their variation can be explained by dose. However, on the basis of reported methodology, we do not find any reason to exclude these results from the subpopulation of studies.

## Discussion

Our systematic analysis revealed that, although individual papers suggested a very good correlation between dose and MN produced, the variation between studies resulted in a poor overall reliability of the assay and suggested that dose alone contributed to between half and two thirds of the variation in the MN measured.

There were two different methods for counting MN following use of the CBMN assay: MNPC and PCMN. The latter has a slightly lower level of interstudy variation than the former, showing a stronger correlation with dose. This may be due to PCMN being a less ambiguous metric. Since the scoring process is binary, with each cell only being scored as positive or negative for MN, erroneous detection of close or overlapping MN will not impact on the result. Finally, PCMN is not affected by MN coalescence, a process whereby several DNA fragments colocate within a single MN. Coalescence of fragments within a cell may result in a similar or even lower number of MN within a cell despite an increase in DNA damage. This is particularly important for higher-LET modalities, where clustered DNA damage results in an increase in short DNA fragments [23, 24].

When analyzing differences in the MN produced per unit of dose, the main variables found to impact the results were particle type, species, and cell type. Other variables such as LET and cyt-B concentration were found to be significant only in extremes and the results were not consistent. Radiation quality is expected to have an impact on MN produced per dose due to differences in DNA double strand break yields and clustering [25]. Species and cell type are also expected to show differences in MN yield due to varying amounts of DNA, radiosensitivity and mutations in the DNA damage response pathway. Despite the evidence that these variables impact on MN production, variations between datasets make the exact differences impossible to evaluate.

One interesting result of the meta-analysis is that MN frequencies are significantly lower in cancer cells than in healthy cells when using the PCMN measure. The reason for this is unknown and more research into this mechanism would be useful. Cancer cells have a reduced DNA repair response meaning DNA damages are less likely to be repaired correctly in comparison to healthy cells. It could be expected that the number of MN would increase as a result of this, however this is the opposite of what was found in this meta-analysis. It could be speculated that MN frequencies are lower in cancer cells because radiation-induced DNA damage is more fatal to them. The reduced repair capacity of cancer cells could potentially result in cells dying before presenting their MN, whereas healthy cells are more likely to survive, allowing their MN to be counted.

A significant finding of this review was the effect of deviation from the CBMN protocol on MN induction. When the protocol timings set out by Fenech in the original and subsequent reviews of the CBMN assay [8, 11] were followed, the variation in the data was reduced in both MN datasets. The two key timings in the CBMN protocol are the addition of cyt-B and the time between cyt-B addition and cell fixation. It is unclear from the data whether one of these timings is more influential on the variation in the data than the other. However, two popular protocols where cyt-B is added immediately after irradiation and the fixation occurs 24 or 48 h following, do not have the same impact on reliability. The addition of cyt-B results in binucleated cells by blocking the second cell division. Early addition may result in the first cell division being blocked with fewer MN produced and late addition could result in multiple cell divisions before blocking, which could increase or decrease MN [11]. Fixation timing may also be significant as around 50% of MN envelopes may rupture within 24 h of mitosis [3].

The subpopulation analysis consists of 58 studies evaluating MNPC and 27 studies evaluating PCMN (34% and 41% of the total studies evaluating the measures, respectively). Controlling the dataset by protocol particle type, cell type, and species showed a large reduction in data variation. The increase in *R*^2^, particularly for the MNPC data (from 0.47 to 0.64), shows that the data can be made more reliable. Although the two measures produce a similar *R*^2^ in the subpopulation, removal of two papers from the PCMN dataset reduces the variation in MN per unit of dose significantly (increase in *R*^2^ from 0.64 to 0.89), and therefore this is still suggested as a better marker for DNA damage. At this point, particle type cannot be fully studied as a factor of variation as there is a significant lack of data, with two or fewer studies available for each of the particles. This finding shows that, although on first impressions MN are a common assay, more focused research following a unified protocol is needed to fully understand their relationship with DNA damage and dose.

One limitation of this paper is that we did not differentiate our results based on scoring procedure (manual or automated microscopy). It has been noted previously that scoring methodology between different labs and individual scorers can result in significantly different MN frequencies [26]. Unfortunately, specific scoring techniques could not be recorded for all papers included in this review as most studies lacked sufficient detail about scoring criteria. This could therefore not be investigated in our analysis. It would be useful to investigate whether the level of automation in MN scoring has an effect, however more data are required before that can be determined.

Phytohemagglutinin (PHA) effect on MN production was also not studied in this analysis. PHA is suggested in the CBMN assay when studying noncycling lymphocytes as it stimulates the cells to divide which is required for MN production. As PHA is not needed in studies which use cycling cells, it was not always used in the various experiments. The effect of its addition on MN is unknown given that noncycling cells cannot produce MN to be used as a comparison.

A final limitation of the lymphocyte data in particular is interindividual radiosensitivity of the donors and the effect this will have on MN induction. A lack of detailed information in individual studies makes this impossible to analyze across the metadata, however some studies found differences in the number of MN induced between donors. Given that full genetic analysis and radiosensitivity were not conducted on each of the volunteers, variation is more likely between samples then when using authenticated cell lines.

## Conclusions

In conclusion, the meta-analysis of all the MN data available showed that variation between studies makes MN a poor predictor of radiation-induced DNA damage. When controlling for protocol, cell type and species in an attempt to create a subpopulation of similar experiments, the resulting data were still too varied to make it possible to predict the exact MN production for a given dose. The measure of PCMN produces more reliable data which can be further focused by controlling for particle type, cell type, and species; therefore, it is recommended that this measure is used for all future studies assessing MN. Limiting the analysis to only papers that follow the Fenech CBMN protocol timings also reduced variation and therefore adhering to the specified timings would yield the most reliable results. By following these recommendations, internal consistency could be improved and therefore cross-examination between studies could establish further effects of radiation modality, cell type, and species on MN induction.

## Supplementary data

Supplementary data is available at *Mutagenesis* online.

Supplementary Table 1. Data from all papers recording MN frequency in response to irradiation with photons.

Supplementary Table 2. Data from all papers recording MN frequency in response to irradiation with protons.

Supplementary Table 3. Data from all papers recording MN frequency in response to irradiation with carbon particles.

Supplementary Table 4. Data from all papers recording MN frequency in response to irradiation with neutrons.

Supplementary Table 5. Data from all papers recording MN frequency in response to irradiation with other particles (alpha, argon, beta, iron, lithium, oxygen, and silicon).

Supplementary Table 6. Data from all papers recording MN frequency in response to irradiation with electrons.

geac001_suppl_Supplementary_Tables
