# Supplementary material for: The suitability of micronuclei as markers of relative biological effect
Source: Mutagenesis. 2022 Feb 8;37(1):3–12. doi: 10.1093/mutage/geac001 (PMC8976228; doi:10.1093/mutage/geac001)
Supplement: geac001_suppl_Supplementary_Tables [file geac001_suppl_supplementary_tables.docx]

Supplementary Tables

The below tables contain details on all 193 papers used for this meta-analysis. Studies that included the effect of two or more particle types are reproduced in all relevant tables.

Table 1., Data from all papers recording MN frequency in response to irradiation with photons.

| **Paper**  *** = Measured MNPC**  **^%^ = Measured PCMN** | **Ref.** | **Particle Type** | **Cell Line** | **Species** | **[Cyt-B]**  **(μg/ml)** | **Time Between Irradiation and Cyt-B Addition**  **(hrs)** | **Time Between Cyt-B Addition and Processing**  **(hrs)** |
| --- | --- | --- | --- | --- | --- | --- | --- |
| Chinhengo., 2020* | (27) | X-ray | DU145  MeWo  Be11  L132 (HeLa) | Human | 2 | 0 | Various (24 – 48) |
| Ghorbanian Kelachayeh., 2020* | (28) | Gamma | HeLa  MRC5 | Human | 4 | 0 | 48 |
| Miszczyk., 2020* | (29) | X-ray | Lymphocyte | Human | 1.2 | 48 | 24 |
| Olofsson., 2020* | (30) | Gamma | U2OS  Lymphocyte | Human | 5.6 | 0 | 48 |
| Pujol-Canadell., 2020* | (31) | Gamma | Lymphocyte | Human | 6 | 44 | 26 |
| Zaguia., 2020* | (32) | Gamma | Lymphocyte | Human | 6 | 48 | 24 |
| Asghari., 2019^%^ | (33) | X-ray | Lymphocyte | Human | 6 | 44 | 28 |
| Babayan., 2019^%^ | (34) | X-ray | MRC5 | Human | 3 | 24 | 48 |
| Barbu., 2019* | (35) | Gamma  X-ray | Lymphocyte | Human | N/P | 44 | 28 |
| Cheng., 2019* | (36) | Gamma | Lymphocyte | Human | 5.6 | 44 | 28 |
| Chevalier., 2019* | (37) | X-ray | SW1353 | Human | 3 | 4 | 22 |
| Francies., 2019* | (38) | X-ray | Lymphocyte | Human | N/P | 23 | 70 |
| Gałecki., 2019* | (39) | Gamma | CHO-K1 | Chinese Hamster | 5.6 | 0 | 22 |
| Ghelishli., 2019^%^ | (40) | X-ray | Lymphocyte | Human | 6 | 44 | 28 |
| Kanagaraj., 2019* | (41) | X-ray | Lymphocyte | Human | 6 | 24 | 48 |
| Karthik., 2019* | (42) | Gamma | Lymphocyte | Human | 6 | 44 | 28 |
| Pouri., 2019^%^ | (43) | X-ray | Lymphocyte | Human | 6 | 44 | 28 |
| Repin., 2019* | (44) | Gamma | Lymphocyte | Human | 6 | 24 | 30 |
| Torabizadeh., 2019^%^ | (45) | X-ray | Lymphocyte | Human | 6 | 44 | 72 |
| Tu., 2019* | (46) | Gamma | CHO-9  EM-C11  XR-C1 | Chinese Hamster | 0.83 | 0 | 20 |
| Wang., 2019* | (47) | X-ray | Hep G2 | Human | 1.5 | 0 | 28 |
| Wang., 2019* | (48) | Gamma | Lymphocyte | Human | 6 | 24 | 44 |
| Zangeneh., 2019^%^ | (49) | X-ray | SKLC-6 | Human | 6 | 24 | 26 |
| Ahmed., 2018*^%^ | (50) | X-ray | Lymphocyte | Human | Not explicitly stated but protocol from (11) is referenced. | | |
| Ariyoshi., 2018* | (51) | X-ray | A603 | Mouse | 2 | 0 | 24 |
| Cartwright., 2018* | (52) | X-ray | CHO | Chinese Hamster | 4 | 22 | N/P |
| Chatterjee., 2018* | (53) | X-ray | Oliogodendrocye progenitor cell | Mouse | 5 | 24 | 0.08 |
| Francies., 2018* | (54) | X-ray | Lymphocyte | Human | N/P | 23 | 70 |
| Pajic., 2018* | (55) | X-ray | Lymphocyte | Human | 3 | 44 | 24 |
| Prevost., 2018* | (56) | X-ray | Dermal Fibroblast | Human | N/P | 24 | 24 |
| Sattarpour., 2018* | (57) | X-ray | Lymphocyte | Human | 6 | 44 | 28 |
| Tian., 2018* | (58) | Gamma | AHH-1 | Human | Not explicity stated but protocol from (59) is referenced. | | |
| Baert., 2017* | (60) | Gamma | Lymphocyte | Human | 6 | 0 | 8 |
| Depuydt., 2017* | (61) | Gamma  X-ray | Lymphocyte | Human | N/P | Various (23 – 44) | Various (28 - 48) |
| Hosseinimehr., 2017* | (62) | X-ray | Lymphocyte | Human | 6 | 44 | 28 |
| Jin., 2017* | (63) | X-ray | MRC-5 | Human | 3 | 2 | 50 |
| Raghuraman., 2017* | (64) | Gamma | CHO | Chinese Hamster | 4 | N/P | 18 |
| Sun., 2017*^%^ | (65) | X-ray | GSC-3 | Human | 2.5 | 0 | 36 |
| Syaifudin., 2017*^%^ | (66) | Gamma | Lymphocyte | Human | 3 | 44 | 28 |
| Weidele., 2017* | (67) | X-ray | Lymphocyte | Human | 6 | 44 | 28 |
| Beinke., 2016*^%^ | (68) | X-ray | Lymphocyte | Human | 6 | 23 | 47 |
| Bertucci., 2016* | (69) | X-ray | Lymphocyte | Human | 6 | 44 | Various (1 & 24) |
| Cheki., 2016* | (70) | X-ray | Lymphocyte | Human | 5 | 44 | 28 |
| Kang., 2016*^%^ | (71) | Gamma | Lymphocyte | Human | 3 | 14 | 29 |
| Lusiyanti., 2016*^%^ | (72) | X-ray | Lymphocyte | Human | 3 | 44 | 28 |
| Nakamura., 2016* | (73) | X-ray | K562 | Human | 6 | Various (6 & 12) | 24 |
| Rostami., 2016* | (74) | X-ray | Lymphocyte | Human | 6 | 44 | 24 |
| Temelie., 2016* | (75) | X-ray | L929 | Mouse | 3 | 5 | 19 |
| Tewari., 2016* | (76) | X-ray | Lymphocyte | Human | N/P | 44 | 28 |
| Tian., 2016* | (77) | Gamma | Lymphocyte | Human | 4.5 | 2 | 24 |
| Vandevoorde., 2016* | (78) | X-ray | T Lymphocyte | Human | 6 | 23 | 49 |
| Wang., 2016* | (79) | X-ray | MC3T3-E1 | Mouse | 2.5 | Various (0, 24 & 48) | 48 |
| Beinke., 2015* | (80) | X-ray | Lymphocyte | Human | 6 | 23 | 47 |
| Francies., 2015* | (81) | X-ray | Lymphocyte | Human | N/P | 23 | 47 |
| Fujisawa., 2015* | (82) | X-ray | U2OS | Human | 2 | 0 | 24 |
| Hosseinimehr., 2015* | (83) | X-ray | Lymphocyte | Human | 6 | 44 | 28 |
| Hou., 2015^%^ | (84) | X-ray | AG01522 | Human | 2.5 | 3 | 48 |
| Litvinchuk., 2015*^%^ | (85) | Gamma | CC-2509 | Human | N/P | 24 | 24 |
| Lusiyanti., 2015* | (86) | Gamma | Lymphocyte | Human | N/P | N/P | N/P |
| Miszczyk., 2015* | (87) | Gamma | Lymphocyte | Human | 6 | 44 | 28 |
| Pajic., 2015* | (88) | X-ray | Lymphocyte | Human | 3 | 44 | 24 |
| Shahani., 2015^%^ | (89) | X-ray | Lymphocyte | Human | 6 | 44 | 28 |
| Shirani., 2015* | (90) | Gamma | CHO-K1 | Chinese Hamster | N/P | N/P | 28 |
| Snijders., 2015* | (91) | Gamma | HaCaT | Human | 3 | 0 | 48 |
| Tamizh Selvan., 2015*^%^ | (92) | Gamma | Lymphocyte | Human | 6 | 44 | 28 |
| Widel., 2015^%^ | (93) | X-ray | HCT116 | Human | 3 | 0 | 48 |
| Alcaraz., 2014* | (94) | X-ray | PNT2 | Human | 6 | 44 | 28 |
| Alcaraz., 2014* | (95) | X-ray | PNT2 | Human | 6 | 44 | 28 |
| Balajee., 2014*^%^ | (96) | Gamma | Lymphocyte | Human | 6 | 44 | 26 |
| Brehwens., 2014* | (97) | X-ray | TK6 | Human | 5.6 | 0 | 24 |
| Chaurasia., 2014* | (98) | X-ray | CHO | Chinese Hamster | 5 | 18 | 0 |
| Cinkilic., 2014* | (99) | X-ray | Lymphocyte | Human | 6 | 44 | 28 |
| Dutta., 2014* | (100) | Gamma | Lymphocyte | Human | 6 | 44 | 28 |
| Leskovac., 2014* | (101) | Gamma | Fibroblast | Human | 2 | 24 | 48 |
| Pei., 2014* | (102) | X-ray | MRC-5 | Human | 15 | 0 | 32 |
| Ryu., 2014*^%^ | (103) | Gamma | Lymphocyte | Human | N/P | 44 | 28 |
| Santos., 2014* | (104) | Gamma | CHO-K1 | Chinese Hamster | 3 | 0 | 48 |
| Selvan., 2014*^%^ | (105) | Gamma | Lymphocyte | Human | 6 | 44 | 28 |
| Seth., 2014* | (106) | Gamma | GM15510  GM15036 | Human | 6 | 4 | 28 |
| Vandersickel., 2014* | (107) | X-ray | Lymphocyte | Human | 6 | 24 | 48 |
| Zhao., 2014* | (108) | Gamma | Lymphocyte | Human | 10 | 40 | 28 |
| Cheong., 2013* | (109) | Gamma | GM15510  GM15036 | Human | 6 | 4 | 28 |
| Ren., 2013* | (110) | Gamma | HMy2.CIR | Human | 3 | 0 | 30 |
| Acharya., 2012*^%^ | (111) | Gamma | Lymphocyte | Human | 3 | 44 | 28 |
| Begum., 2012*^%^ | (112) | Gamma | Lymphocyte | Human | 6 | 44 | 28 |
| Heshmati., 2012* | (113) | X-ray | HeLa  MRC5 | Human | 4 | 24 | 30 |
| Schmid., 2012*^%^ | (114) | X-ray | A_L_ | Chinese Hamster | 10 | 0 | 24 |
| Staaf., 2012*^%^ | (115) | X-ray | Lymphocyte | Human | 5.56 | N/P | N/P |
| Zuo., 2012* | (116) | Gamma | HL7702 | Human | 4 | N/P | 18 |
| Aypar., 2011^%^ | (117) | X-ray | GM10115 | Chinese Hamster/Human | 2 | 2 | 36 |
| Belloni., 2011* | (118) | X-ray | Lymphocyte | Human | 6 | 44 | 28 |
| Hosseinimehr., 2011* | (119) | Gam(113)ma | Lymphocyte | Human | 6 | 44 | 28 |
| Konopacka., 2011^%^ | (120) | Photon | A549  BEAS-2B  NHDF | Human | 2 | 0 | 48 |
| Kalpana., 2010* | (121) | X-ray | Lymphocyte | Human | N/P | 44 | 28 |
| Schmid., 2010*^%^ | (122) | X-ray | EpiDermFT | Human | 3 | 0 | 48 |
| Vandersickel., 2010* | (123) | X-ray | MCF10A | Human | 2.25 | 0 | 48 |
| Vandersickel., 2010* | (124) | Gamma | MCF10A | Human | 2.25 | 2 | 46 |
| Acharya., 2009*^%^ | (125) | Gamma | Lymphocyte | Human | 5 | 44 | 24 |
| Du., 2009^%^ | (126) | Gamma | MEF | Mouse | 4 | Various (24 & 72) | 48 |
| Jingyin., 2009^%^ | (127) | Gamma | A7  M2 | Human | 2 | 24 | 24 |
| Kalpana., 2009* | (128) | Gamma | Lymphocyte | Human | 3 | 44 | 28 |
| Ryabokon., 2009^%^ | (129) | Gamma | Lymphocyte | Human | 6 | 48 | 24 |
| Singh., 2009* | (130) | Gamma | Lymphocyte | Human | 3 | 44 | 28 |
| Devipriya., 2008* | (131) | Gamma | Lymphocyte | Human | 0.6 | 44 | 28 |
| Kim., 2008* | (132) | Gamma | HL-60 | Human | 3 | N/P | 24 |
| Wojewódzka., 2008^%^ | (133) | X-ray | T Lymphocyte  B Lymphocyte | Human | 5.6 | Various (48 & 72) | 24 |
| Zhang., 2008^%^ | (134) | Gamma | AG1522 | Human | 2.5 | 5 | 48 |
| Gangopadhyay., 2007^%^ | (135) | Gamma | Lymphocyte | Human | 3 | 44 | 28 |
| Groesser., 2007* | (136) | X-ray | MCF10A  CHO-Xrs-6  V79 | Human  Chinese Hamster | 3 | 0 | 24 |
| Kim., 2007* | (137) | Gamma | CCD 18Lu | Human | 3 | 0 | 24 |
| Mozdarani., 2007*^%^ | (138) | Gamma | Lymphocyte | Human | 6 | 28 | 44 |
| Pathak., 2007* | (139) | Gamma | V79 | Chinese Hamster | 3 | 0 | 20 |
| Pathak., 2007* | (140) | Gamma | V79  M5 | Chinese Hamster | 3 | 0 | 20 |
| Del Baño., 2006* | (141) | Gamma | Lymphocyte | Human | 3 | 44 | 28 |
| Hori., 2006* | (142) | Gamma | RAW264.7 | Mouse | 3 | N/P | 24 |
| Lehnert., 2006*^%^ | (143) | X-ray | MCF-12A | Human | 1.5 | 0 | 48 |
| Prasad., 2006* | (144) | Gamma | Lymphocyte | Human | 0.6 | 44 | 28 |
| Srinivasan., 2006* | (145) | Gamma | Lymphocyte | Human | 0.6 | 44 | 28 |
| Jagetia., 2005*^%^ | (146) | Gamma | Lymphocyte | Human | 5 | 44 | 28 |
| Rithidech., 2005* | (147) | Gamma | Lymphocyte | Human | 3 | 44 | 28 |
| Somodi., 2005^%^ | (148) | X-ray | CHO-9  XR-C1  EM-C11 | Chinese Hamster | 1.2 | 0 | 16 |
| Yang., 2005^%^ | (149) | X-ray | AGO1522 | Human | 1.5 | 0 | 72 |
| Akudugu., 2004* | (150) | Gamma | G-44  G-112  G-60  G-120  Be11  BPH-1  1535T  SCC 4451  1542N | Human | 2 | 0 | 44 |
| Konopacka., 2004* | (151) | X-ray | Lymphocyte | Human | 6 | 44 | 28 |
| Lee., 2004*^%^ | (152) | Gamma | Lymphocyte | Human | 6 | 68 | 28 |
| Slowinski., 2004* | (153) | Gamma | LN-405  8-MG-BA  GAMG  DK-MG  DBTRG-05-MG  GOS-3  42-MG-BA  U-138-MG | Human | 2 | 0 | 72 |
| Bhat., 2003*^%^ | (154) | Gamma | Lymphocyte | Human | 5 | 44 | 23 |
| Jagetia., 2003^%^ | (155) | Gamma | Lymphocyte | Human | 5 | 44 | 28 |
| Jagetia., 2003^%^ | (156) | Gamma | HeLa | Human | 3 | 6 | Various (14, 24 & 34) |
| Słonina., 2003*^%^ | (157) | X-ray | HEKn  HFIB  3T3 | Human  Mouse | Various (1.5 & 2) | 0 | Various (48 & 72) |
| Thomas., 2003* | (158) | Gamma | Lymphocyte | Human | 4.5 | 44 | 26 |
| Jagetia., 2002*^%^ | (159) | Gamma | Lymphocyte | Human | 5 | 44 | 28 |
| Müller., 2002* | (160) | X-ray | Lymphocyte | Human | 5 | 44 | 32 |
| Schäfer., 2002^%^ | (161) | X-ray | TK6  TKE6  WTK1 | Human | 3 | Various (0 & 4) | 24 |
| Eastham., 2001* | (162) | Gamma | SiHa  778  MS751  C33a  HeLa  CaSki  HT3  C41 | Human | 2 | 6 | 42 |
| Ponsa., 2001*^%^ | (163) | Gamma | Lymphocyte | Human | 6 | 44 | 28 |
| Sgura., 2001* | (164) | X-ray | HFFF2 | Human | 3 | 0 | 24 |
| Shao., 2001* | (165) | X-ray | HSG | Human | 3 | 30 | 24 |
| Vral., 2001* | (166) | Gamma | T Lymphocyte  B Lymphocyte | Human | 6 | 42 | 30 |
| Yoshida., 2001* | (167) | Gamma | 143B  KT-1 | Human | 0.5 | 0 | Various (24 & 48) |
| Bishay., 2000* | (168) | Gamma | Lymphoblast | Human | 5 | 0 | 24 |
| He., 2000*^%^ | (169) | X-ray | Lymphocyte | Human | 4.5 | 44 | 28 |
| Jagetia., 2000*^%^ | (170) | Gamma | V79 | Chinese Hamster | 3 | 4 | Various (12, 18 & 24) |
| Joksic., 2000*^%^ | (171) | X-ray | Lymphocyte | Human | 6 | 48 | 24 |
| Adiga., 1999*^%^ | (172) | Gamma | V79 | Chinese Hamster | 3 | 6 | Various (10, 16 & 22) |
| Kriehuber., 1999* | (173) | X-ray | SCL-II | Human | 1.2 | 30 | 24 |
| Mariya., 1999* | (174) | X-ray | PECA4197 | Human | 1.5 | 0 | 48 |
| Guo., 1998* | (175) | X-ray | CHO-K1  SHIN-3  DU-145  F9  Colo 320 DM | Chinese Hamster  Human  Mouse | 0.5 | 0 | 48 |
| Keshava., 1998^%^ | (176) | X-ray | V79 | Chinese Hamster | 3 | 0 | 16 |
| Takagi., 1998* | (177) | X-ray | HT-1080 | Human | 1.5 | Various (0 & 24) | 50 |
| Vral., 1998* | (178) | Gamma | T Lymphocyte  B Lymphocyte | Human | 3.5 | Various (42 & 65) | Various (28 & 55) |
| Wuttke., 1998* | (179) | X-ray | Lymphocyte | Human | 5 | 44 | 25 |
| Catena., 1997*^%^ | (180) | X-ray | Lymphocyte | Human  Horse | 3 | 48 | 24 |
| Kim., 1997* | (181) | Gamma | Lymphocyte | Mouse  Rabbit  Goat  Human | 4 | Various (21 & 44) | Various (22 & 28) |
| Manti., 1997*^%^ | (182) | X-ray | V79-379A | Chinese Hamster | 0.5 | 4 | 18 |
| Paul., 1997*^%^ | (183) | Gamma | Lymphocyte | Human | 3 | 44 | 28 |
| Paul., 1997*^%^ | (184) | Gamma | Lymphocyte | Human | 3 | 44 | 28 |
| Vral., 1997* | (185) | Gamma | Lymphocyte | Human | 3.5 | 42 | 28 |
| Darroudi., 1996*^%^ | (186) | X-ray | Lymphocyte  Hep G2 | Human | 6 | 44 | 28 |
| Gajdusek., 1996* | (187) | Gamma | Smooth Muscle | Rat | 2 | 0 | 120 |
| Gaziev., 1996* | (188) | Gamma | Lymphocyte | Human | 5 | 44 | 28 |
| Keshava., 1996^%^ | (189) | X-ray | V79 | Chinese Hamster | 3 | 0 | 16 |
| Köksal., 1996*^%^ | (190) | Gamma | Lymphocyte | Human | 6 | 48 | 20 |
| Mill., 1996* | (191) | X-ray  Photon | Lymphocyte | Human | 3 | 44 | 28 |
| Vijayalaxmi., 1996*^%^ | (192) | Gamma | Lymphocyte | Human | 4 | 44 | 28 |
| Courdi., 1995* | (193) | Gamma | CAL4 | Human | 2 | 0 | 48 |
| Slavotinek., 1995* | (194) | X-ray | RABI  NV12  JEOLI  CV123  CV477  TEMAR  CV56  ANDMA | N/P | 2 | 0 | Various (24, 30, 36, 42 & 48) |
| Vijayalaxmi., 1995* | (195) | X-ray  Gamma | Lymphocyte | Human | 4 | 0 | 24 |
| Catena., 1994*^%^ | (196) | X-ray | Lymphocyte | Human  Dog | 3 | 48 | 24 |
| Ono., 1994* | (197) | Gamma | SCVII | Mouse | 2 | N/P | Various |
| Silva., 1994*^%^ | (198) | Gamma | Lymphocyte | Human | Various | Various | Various |
| Verhaegen., 1994* | (199) | X-ray  Gamma | Lymphocyte | Human | 3.5 | 42 | 28 |
| Bush., 1993* | (200) | Gamma | RT112  MGH-U1  D283MED  RT112  MGH-U1  HX142  D283MED | Human | Various (0.5, 1, 1.5 & 2) | 2 | Various (24, 48, 72, 96, 120 & 144) |
| Hurwitz., 1993* | (201) | Gamma | CHO AUXB1 | Chinese Hamster | 3 | 6 | 12 |
| Littlefield., 1993* | (202) | X-ray | Lymphocyte | Human | 6 | 42 | 24 |
| Slavotinek., 1993* | (203) | X-ray | RABI  TEMAR  LICR-LON  DEW1  X63  K562 | Human | 2 | 0 | Various (12, 24, 30 & 36) |
| Darroudi., 1992* | (204) | X-ray | Splenocyte | Mouse | 6 | 24 | 26 |
| Armitage., 1991* | (205) | X-ray | T24  SV-HUC-1  NT11  BC16 | Human |  |  |  |
| Balasem., 1991*^%^ | (206) | Gamma | Lymphocyte | Human | 3 | 44 | 28 |
| Gantenberg., 1991* | (207) | X-ray | Lymphocyte | Human | 5 | 44 | N/P |
| Odagiri., 1990* | (208) | X-ray | Lymphocyte | Human | 3 | 44 | 28 |
| Erexson., 1989^%^ | (209) | Gamma | Lymphocyte  Mononuclear Leukocyte | Mouse | 3 | 21 | 29 |
| Koksal., 1989*^%^ | (210) | X-ray | Lymphocyte | Human | 3 | Various (16, 24, 32 & 44) | Various (4, 12, 16, 20, 24, 28, 32, 40, 48 & 56) |
| Kormos., 1988*^%^ | (211) | X-ray | Lymphocyte | Human | 3 | 44 | 28 |
| Prosser., 1988*^%^ | (212) | X-ray | Lymphocyte | Human | Various (1.5, 3, 6 & 15) | 44 | 28 |
| Ramalho., 1988* | (213) | X-ray | Lymphocyte | Human | 3 | 44 | 28 |
| Fenech., 1985* | (8) | X-ray | Lymphocyte | Human | 3 | 44 | 28 |

N/P = Not published

**Table 2., Data from all papers recording MN frequency in response to irradiation with protons.**

| Paper  *** = Measured MNPC**  ^%^ = Measured PCMN | Ref. | Proton Particle Energy  (MeV) | Proton Particle LET  (keV/μm) | Cell Line | Species | [Cyt-B]  (μg/ml) | Time Between Irradiation and Cyt-B Addition  (hrs) | Time Between Cyt-B Addition and Processing  (hrs) |
| --- | --- | --- | --- | --- | --- | --- | --- | --- |
| Miszczyk., 2020* | (29) | 60 | 2.9 | Lymphocyte | Human | 1.2 | 48 | 24 |
| Litvinchuk., 2015*^%^ | (85) | 30 | 1.9 | CC-2509 | Human | N/P | 24 | 24 |
| Miszczyk., 2015* | (87) | 60 | 1.05 | Lymphocyte | Human | 6 | 44 | 28 |
| Schmid., 2012*^%^ | (114) | 20 | 2.65 | A_L_ | Chinese Hamster | 10 | 0 | 24 |
| Go., 2011* | (214) | 50 | 34.6 | Lymphocyte | Human | 3 | 44 | 28 |
| Schmid., 2010*^%^ | (122) | 20 | 2.66 | EpiDermFT | Human | 3 | 0 | 48 |
| Sgura., 2001* | (164) | 6  3 | 7.7  28.5 | HFFF2 | Human | 3 | 0 | 24 |
| Joksic., 2000*^%^ | (171) | 22.6 | 2.31 | Lymphocyte | Human | 6 | 48 | 24 |

N/P = Not published

Table 3., Data from all papers recording MN frequency in response to irradiation with carbon particles.

| **Paper**  *** = Measured MNPC**  **^%^ = Measured PCMN** | **Ref.** | **Carbon Particle Energy**  **(MeV)** | **Carbon Particle LET**  **(keV/μm)** | **Cell Line** | **Species** | **[Cyt-B]**  **(μg/ml)** | **Time Between Irradiation and Cyt-B Addition**  **(hrs)** | **Time Between Cyt-B Addition and Processing**  **(hrs)** |
| --- | --- | --- | --- | --- | --- | --- | --- | --- |
| Buglewicz., 2019* | (215) | 290 | 14  20  31  43 | CHO | Chinese Hamster | N/P | N/P | N/P |
| Chevalier., 2019* | (37) | 289 | 73 | SW1353 | Human | 3 | 4 | 22 |
| Cartwright., 2018* | (52) | 290 | 13  50 | CHO | Chinese Hamster | 4 | 22 | N/P |
| Prevost., 2018* | (56) | 75 | 30.3 | Dermal Fibroblast | Human | N/P | 24 | 24 |
| Sun., 2017*^%^ | (65) | 1700.15 | 17.96 | GSC-3 | Human | 2.5 | 0 | 36 |
| Fujisawa., 2015* | (82) | 3480 | 70 | U2OS | Human | 2 | 0 | 24 |
| Pei., 2014* | (102) | N/P | N/P | MRC-5 | Human | 15 | 0 | 32 |
| Schmid., 2012*^%^ | (114) | 55 | 310 | A_L_ | Chinese Hamster / Human | 10 | 0 | 24 |
| Pathak., 2007* | (139) | 57.24 | 295 | V79 | Chinese Hamster | 3 | 0 | 20 |
| Shao., 2007* | (216) | 290 | 100 | HSG | Human | 2 | 3 | 26 |
| Shao., 2001* | (165) | 290 | 100 | HSG | Human | 3 | 30 | 24 |

N/P = Not published

Table 6., Data from all papers recording MN frequency in response to irradiation with electrons.

| **Paper**  *** = Measured MNPC**  **^%^ = Measured PCMN** | **Ref.** | **Electron Particle Energy**  **(MeV)** | **Electron Particle LET**  **(keV/μm)** | **Cell Line** | **Species** | **[Cyt-B]**  **(μg/ml)** | **Time Between Irradiation and Cyt-B Addition**  **(hrs)** | **Time Between Cyt-B Addition and Processing**  **(hrs)** |
| --- | --- | --- | --- | --- | --- | --- | --- | --- |
| Babayan., 2019^%^ | (34) | 3.6 | 0.19 | MRC5 | Human | 3 | 24 | 48 |
| Acharya., 2012*^%^ | (111) | 8 | 0.21 | Lymphocyte | Human | 3 | 44 | 28 |
| Konopacka., 2011^%^ | (120) | 22 | 0.25 | A549  BEAS-2B  NHDF | Human | 2 | 0 | 48 |
| Acharya., 2009*^%^ | (125) | 8 | 0.21 | Lymphocyte | Human | 5 | 44 | 24 |

N/P = Not published

Table 5., Data from all papers recording MN frequency in response to irradiation with neutrons.

| **Paper**  *** = Measured MNPC**  **^%^ = Measured PCMN** | **Ref.** | **Neutron Particle Energy**  **(MeV)** | **Neutron Particle LET**  **(keV/μm)** | **Cell Line** | **Species** | **[Cyt-B]**  **(μg/ml)** | **Time Between Irradiation and Cyt-B Addition**  **(hrs)** | **Time Between Cyt-B Addition and Processing**  **(hrs)** |
| --- | --- | --- | --- | --- | --- | --- | --- | --- |
| Pujol-Canadell., 2020* | (31) | N/P | N/P | Lymphocyte | Human | 6 | 44 | 26 |
| Vandersickel., 2014* | (107) | 29 | 20 | Lymphocyte | Human | 6 | 24 | 48 |
| Cheong., 2013* | (109) | N/P | N/P | GM15510  GM15036 | Human | 6 | 4 | 28 |
| Vandersickel., 2010* | (123) | 29 | 20 | MCF10A | Human | 2.25 | 0 | 48 |
| Vral., 2001* | (166) | 5.5 | 20 | B Lymphocyte  T Lymphocyte | Human | 6 | 42 | 30 |
| Wuttke., 1998* | (179) | 6 | N/P | Lymphocyte | Human | 5 | 44 | 25 |

N/P = Not published

Table 5., Data from all papers recording MN frequency in response to irradiation with other particles (alpha, argon, beta, iron, lithium, oxygen and silicon).

| Paper  *** = Measured MNPC**  ^%^ = Measured PCMN | Ref. | Particle Type | Particle Energy  (MeV) | Particle LET  (keV/μm) | Cell Line | Species | [Cyt-B]  (μg/ml) | Time Between Irradiation and Cyt-B Addition  (hrs) | Time Between Cyt-B Addition and Processing  (hrs) |
| --- | --- | --- | --- | --- | --- | --- | --- | --- | --- |
| Kanagaraj., 2019* | (41) | Alpha | 3.86 | N/P | Lymphocyte | Human | 6 | 24 | 48 |
| Karthik., 2019* | (42) | Alpha | 5.49 | 87.03 | Lymphocyte | Human | 6 | 44 | 28 |
| Ren., 2013* | (110) | Alpha | 5.48 | 100 | HMy2.CIR | Human | 3 | N/P | 48 |
| Kalanxhi., 2012* | (217) | Alpha | 5.59 | 85.65 | MCF-7 | Human | 2 | N/P | N/P |
| Staaf., 2012*^%^ | (115) | Alpha | 4.6 | 97.4 | Lymphocyte | Human | 5.56 | N/P | N/P |
| Shao., 2008* | (218) | Alpha | 8 | 100 | HCF7  MDAMB231 | Human | 1 | 24 | 48 |
| Manti., 1997*^%^ | (182) | Alpha | 3.9 | 118 | V79-379A | Chinese Hamster | 0.5 | 4 | 18 |
| Mill., 1996* | (191) | Alpha | 35  30 | 20  23 | Lymphocyte | Human | 3 | 44 | 28 |
| Nelson., 1996* | (219) | Alpha | 3.2 | 124 | CHO | Chinese Hamster | 3 | 0 | 0.13 |
| Courdi., 1995* | (193) | Argon | 7.1 | 1590 | CAL4 | Human | 2 | 0 | 48 |
| Mill., 1996* | (191) | Beta | 2.27 | N/P | Lymphocyte | Human | 3 | 44 | 28 |
| Snijders., 2015* | (91) | Iron | 0.3  1 | 237  151 | HaCaT | Human | 3 | 0 | 48 |
| Aypar., 2011^%^ | (117) | Iron | 56000 | 150 | GM10115 | Chinese Hamster | 2 | 2 | 36 |
| Groesser., 2007* | (136) | Iron | 1 | 151 | V79 | Chinese Hamster | 3 | 0 | 24 |
| Pathak., 2007* | (139) | Lithium | 45 | 60 | V79 | Chinese Hamster | 3 | 0 | 20 |
| Pathak., 2007* | (140) | Lithium | 45 | 60 | V79 | Chinese Hamster | 3 | 0 | 20 |
| Pathak., 2007* | (139) | Oxygen | 58 | 625 | V79 | Chinese Hamster | 3 | 0 | 20 |
| Snijders., 2015* | (91) | Silicon | 0.3  1 | 68  44 | HaCaT | Human | 3 | 0 | 48 |

References

27. Chinhengo A, Serafin, A., Akudugu, J. Radiosensitization by Radiofrequency Fields is Correlated with Micronucleus Yield and Proliferative Index. Plasma Medicine. 2020;10:27-43.

28. Kelachayeh SG, Sangtarash, M.H., Mozdarani, H. The effects of melatonin on the frequency of micronuclei induced by ionizing radiation in cancerous and normal cell lines. Iranian Journal of Radiation Research. 2020;18:57-64.

29. Miszczyk J, Rawojc K. Effects of culturing technique on human peripheral blood lymphocytes response to proton and X-ray radiation. Int J Radiat Biol. 2020;96(4):424-33.

30. Olofsson D, Cheng L, Fernandez RB, Plodowska M, Riego ML, Akuwudike P, et al. Biological effectiveness of very high gamma dose rate and its implication for radiological protection. Radiat Environ Biophys. 2020;59(3):451-60.

31. Pujol-Canadell M, Perrier JR, Cunha L, Shuryak I, Harken A, Garty G, et al. Cytogenetically-based biodosimetry after high doses of radiation. PLoS One. 2020;15(4):e0228350.

32. Zaguia N, Laplagne E, Colicchio B, Cariou O, Al Jawhari M, Heidingsfelder L, et al. A new tool for genotoxic risk assessment: Reevaluation of the cytokinesis-block micronucleus assay using semi-automated scoring following telomere and centromere staining. Mutat Res. 2020;850-851:503143.

33. Asghari M, Shaghaghi Z, Farzipour S, Ghasemi A, Hosseinimehr SJ. Radioprotective effect of olanzapine as an anti-psychotic drug against genotoxicity and apoptosis induced by ionizing radiation on human lymphocytes. Mol Biol Rep. 2019;46(6):5909-17.

34. Babayan N, Grigoryan B, Khondkaryan L, Tadevosyan G, Sarkisyan N, Grigoryan R, et al. Laser-Driven Ultrashort Pulsed Electron Beam Radiation at Doses of 0.5 and 1.0 Gy Induces Apoptosis in Human Fibroblasts. Int J Mol Sci. 2019;20(20).

35. Barbu L, Obreja, D., Duliu, O.G., Celarel, A., Cenusa, C. The cell micronuclei response to ionizing radiation in the case of gamma and x-ray exposure. Romanian Journal of Physics. 2019.

36. Cheng L, Brzozowska-Wardecka B, Lisowska H, Wojcik A, Lundholm L. Impact of ATM and DNA-PK Inhibition on Gene Expression and Individual Response of Human Lymphocytes to Mixed Beams of Alpha Particles and X-Rays. Cancers (Basel). 2019;11(12).

37. Chevalier F, Hamdi DH, Lepleux C, Temelie M, Nicol A, Austry JB, et al. High LET Radiation Overcomes In Vitro Resistance to X-Rays of Chondrosarcoma Cell Lines. Technol Cancer Res Treat. 2019;18:1533033819871309.

38. Francies FZ, Herd O, Cairns A, Nietz S, Murdoch M, Slabbert J, et al. Chromosomal radiosensitivity of triple negative breast cancer patients. Int J Radiat Biol. 2019;95(11):1507-16.

39. Galecki M, Tartas A, Szymanek A, Sims E, Lundholm L, Sollazzo A, et al. Precision of scoring radiation-induced chromosomal aberrations and micronuclei by unexperienced scorers. Int J Radiat Biol. 2019;95(9):1251-8.

40. Ghelishli N, Ghasemi A, Hosseinimehr SJ. The Influence of Piperine on the Radioprotective Effect of Curcumin in Irradiated Human Lymphocytes. Turk J Pharm Sci. 2019;16(3):366-70.

41. Kanagaraj K, Rajan V, Pandey BN, Thayalan K, Venkatachalam P. Primary and secondary bystander effect and genomic instability in cells exposed to high and low linear energy transfer radiations. Int J Radiat Biol. 2019;95(12):1648-58.

42. Karthik K, Rajan V, Pandey BN, Sivasubramanian K, Paul SFD, Venkatachalam P. Direct and bystander effects in human blood lymphocytes exposed to (241)Am alpha particles and the relative biological effectiveness using chromosomal aberration and micronucleus assay. Int J Radiat Biol. 2019;95(6):725-36.

43. Pouri M, Shaghaghi Z, Ghasemi A, Hosseinimehr SJ. Radioprotective Effect of Gliclazide as an Anti-Hyperglycemic Agent Against Genotoxicity Induced by Ionizing Radiation on Human Lymphocytes. Cardiovasc Hematol Agents Med Chem. 2019;17(1):40-6.

44. Repin M, Pampou S, Garty G, Brenner DJ. RABiT-II: A Fully-Automated Micronucleus Assay System with Shortened Time to Result. Radiat Res. 2019;191(3):232-6.

45. Torabizadeh SA, Rezaeifar M, Jomehzadeh A, Nabizadeh Haghighi F, Ansari M. Radioprotective Potential of Sulindac Sulfide to Prevent DNA Damage Due to Ionizing Radiation. Drug Des Devel Ther. 2019;13:4127-34.

46. Tu W, Dong C, Fu J, Pan Y, Kobayashi A, Furusawa Y, et al. Both irradiated and bystander effects link with DNA repair capacity and the linear energy transfer. Life Sci. 2019;222:228-34.

47. Wang X, Tu W, Chen D, Fu J, Wang J, Shao C, et al. Autophagy suppresses radiation damage by activating PARP-1 and attenuating reactive oxygen species in hepatoma cells. Int J Radiat Biol. 2019;95(8):1051-7.

48. Wang Q, Rodrigues MA, Repin M, Pampou S, Beaton-Green LA, Perrier J, et al. Automated Triage Radiation Biodosimetry: Integrating Imaging Flow Cytometry with High-Throughput Robotics to Perform the Cytokinesis-Block Micronucleus Assay. Radiat Res. 2019;191(4):342-51.

49. Zangeneh M, Nedaei HA, Mozdarani H, Mahmoudzadeh A, Salimi M. Enhanced cytotoxic and genotoxic effects of gadolinium-doped ZnO nanoparticles on irradiated lung cancer cells at megavoltage radiation energies. Mater Sci Eng C Mater Biol Appl. 2019;103:109739.

50. Ahmed MM, Said, Z.S., Montaser, S.A., El-Tawil, G.A. Antioxidant and antimutagenic properties of calcium sennosides in γ-Irradiated human blood cultures. Int J Radiat Res 2018. 2018;16(3):323-32.

51. Ariyoshi K, Miura T, Kasai K, Akifumi N, Fujishima Y, Yoshida MA. Radiation-induced bystander effect in large Japanese field mouse (Apodemus speciosus) embryonic cells. Radiat Environ Biophys. 2018;57(3):223-31.

52. Cartwright IM, Su C, Haskins JS, Salinas VA, Sunada S, Yu H, et al. DNA Repair Deficient Chinese Hamster Ovary Cells Exhibiting Differential Sensitivity to Charged Particle Radiation under Aerobic and Hypoxic Conditions. Int J Mol Sci. 2018;19(8).

53. Chatterjee J, Nairy RK, Langhnoja J, Tripathi A, Patil RK, Pillai PP, et al. ER stress and genomic instability induced by gamma radiation in mice primary cultured glial cells. Metab Brain Dis. 2018;33(3):855-68.

54. Francies FZ, Wainwright R, Poole J, De Leeneer K, Coene I, Wieme G, et al. Diagnosis of Fanconi Anaemia by ionising radiation- or mitomycin C-induced micronuclei. DNA Repair (Amst). 2018;61:17-24.

55. Pajic J, Rovcanin B, Kekic D, Jovicic D, Milovanovic APS. The influence of redox status on inter-individual variability in the response of human peripheral blood lymphocytes to ionizing radiation. Int J Radiat Biol. 2018;94(6):569-75.

56. Prevost V, Sichel F, Pottier I, Leduc A, Lagadu S, Laurent C. Production of early and late nuclear DNA damage and extracellular 8-oxodG in normal human skin fibroblasts after carbon ion irradiation compared to X-rays. Toxicol In Vitro. 2018;52:116-21.

57. Sattarpour Z, Baradaran B, Farajollahi A, Asghari Jafarabadi M, Khazeh V, Pirayesh Islamian J. Evaluation of an Immunomodulator Drug as a Radioprotectant on Human Peripheral Blood Lymphocytes In Vitro. Middle East Journal of Cancer. 2018;9(1):35-40.

58. Tian XL, Lu X, Feng JB, Cai TJ, Li S, Tian M, et al. Alterations in histone acetylation following exposure to (60)Co gamma-rays and their relationship with chromosome damage in human lymphoblastoid cells. Radiat Environ Biophys. 2018;57(3):215-22.

59. Fenech M. The lymphocyte cytokinesis-block micronucleus cytome assay and its application in radiation biodosimetry. Health Phys. 2010;98(2):234-43.

60. Baert A, Depuydt J, Van Maerken T, Poppe B, Malfait F, Van Damme T, et al. Analysis of chromosomal radiosensitivity of healthy BRCA2 mutation carriers and non-carriers in BRCA families with the G2 micronucleus assay. Oncol Rep. 2017;37(3):1379-86.

61. Depuydt J, Baeyens A, Barnard S, Beinke C, Benedek A, Beukes P, et al. RENEB intercomparison exercises analyzing micronuclei (Cytokinesis-block Micronucleus Assay). Int J Radiat Biol. 2017;93(1):36-47.

62. Hosseinimehr SJ, Fathi M, Ghasemi A, Shiadeh SN, Pourfallah TA. Celecoxib mitigates genotoxicity induced by ionizing radiation in human blood lymphocytes. Res Pharm Sci. 2017;12(1):82-7.

63. Jin C, Liu, H., Li, W., Cao, R. Biological effects of human lung cells MRC-5 in CBCT positioning for image-guided radiotherapy. Nuclear Science and Techniques. 2017;28.

64. Raghuraman M, Verma P, Kunwar A, Phadnis PP, Jain VK, Priyadarsini KI. Cellular evaluation of diselenonicotinamide (DSNA) as a radioprotector against cell death and DNA damage. Metallomics. 2017;9(6):715-25.

65. Sun F, Zhang, X., Zhou, X., Hua, J., Zhang, Y., Wang, B., Hu, W., Ding, N., He, J., Li, H., Pei, H., Zhao, X., Zhou, G., Wang, J. Comparisons between bio-radiation effects of X-rays and carbon-ion irradiation on glioma stem cells. Int J Clin Exp Med. 2017;10:4639-48.

66. Syaifudin M, Lusiyanti, Y., Purnami, S., Lee, Y.S., Kang, C.M. Assessment of ionizing radiation induced dicentric chromosome and micronuclei in human Peripheral blood lymphocytes for preliminary reconstruction of cytogenetic biodosimetry. Atom Indonesia. 2017;43(1):47-54.

67. Weidele K, Beneke S, Burkle A. The NAD(+) precursor nicotinic acid improves genomic integrity in human peripheral blood mononuclear cells after X-irradiation. DNA Repair (Amst). 2017;52:12-23.

68. Beinke C, Port M, Riecke A, Ruf CG, Abend M. Adaption of the Cytokinesis-Block Micronucleus Cytome Assay for Improved Triage Biodosimetry. Radiat Res. 2016;185(5):461-72.

69. Bertucci A, Smilenov LB, Turner HC, Amundson SA, Brenner DJ. In vitro RABiT measurement of dose rate effects on radiation induction of micronuclei in human peripheral blood lymphocytes. Radiat Environ Biophys. 2016;55(1):53-9.

70. Cheki M, Shirazi A, Mahmoudzadeh A, Bazzaz JT, Hosseinimehr SJ. The radioprotective effect of metformin against cytotoxicity and genotoxicity induced by ionizing radiation in cultured human blood lymphocytes. Mutat Res. 2016;809:24-32.

71. Kang CM, Yun HJ, Kim H, Kim CS. Strong Correlation among Three Biodosimetry Techniques Following Exposures to Ionizing Radiation. Genome Integr. 2016;7:11.

72. Lusiyanti Y, Alatas Z, Syaifudin M, Purnami S. Establishment of a Dose-response Curve for X-ray-Induced Micronuclei in Human Lymphocytes. Genome Integr. 2016;7:7.

73. Nakamura A, Monzen S, Takasugi Y, Wojcik A, Mariya Y. Application of cell sorting for enhancing the performance of the cytokinesis-block micronucleus assay. J Radiat Res. 2016;57(2):121-6.

74. Rostami A, Moosavi SA, Changizi V, Abbasian Ardakani A. Radioprotective effects of selenium and vitamin-E against 6MV X-rays in human blood lymphocytes by micronucleus assay. Med J Islam Repub Iran. 2016;30:367.

75. Temelie M, Stroe D, Petcu I, Mustaciosu C, Moisoi N, Savu D. Bystander effects and compartmental stress response to X-ray irradiation in L929 cells. Radiat Environ Biophys. 2016;55(3):371-9.

76. Tewari S, Khan K, Husain N, Rastogi M, Mishra SP, Srivastav AK. Peripheral Blood Lymphocytes as In Vitro Model to Evaluate Genomic Instability Caused by Low Dose Radiation. Asian Pac J Cancer Prev. 2016;17(4):1773-7.

77. Tian XL, Zhao H, Cai TJ, Lu X, Chen DQ, Li S, et al. Dose-effect relationships of nucleoplasmic bridges and complex nuclear anomalies in human peripheral lymphocytes exposed to 60Co gamma-rays at a relatively low dose. Mutagenesis. 2016;31(4):425-31.

78. Vandevoorde C, Vral A, Vandekerckhove B, Philippe J, Thierens H. Radiation Sensitivity of Human CD34(+) Cells Versus Peripheral Blood T Lymphocytes of Newborns and Adults: DNA Repair and Mutagenic Effects. Radiat Res. 2016;185(6):580-90.

79. Wang C, Blough E, Dai X, Olajide O, Driscoll H, Leidy JW, et al. Protective Effects of Cerium Oxide Nanoparticles on MC3T3-E1 Osteoblastic Cells Exposed to X-Ray Irradiation. Cell Physiol Biochem. 2016;38(4):1510-9.

80. Beinke C, Port M, Abend M. Automatic versus manual lymphocyte fixation: impact on dose estimation using the cytokinesis-block micronucleus assay. Radiat Environ Biophys. 2015;54(1):81-90.

81. Baeyens A. Chromosomal radiosensitivity of lymphocytes in South African breast cancer patients of different ethnicity: An indirect measure of cancer susceptibility. S Afr Med J. 2015;105(8):675-8.

82. Fujisawa H, Nakajima NI, Sunada S, Lee Y, Hirakawa H, Yajima H, et al. VE-821, an ATR inhibitor, causes radiosensitization in human tumor cells irradiated with high LET radiation. Radiat Oncol. 2015;10:175.

83. Hosseinimehr SJ, Ghaffari-Rad V, Rostamnezhad M, Ghasemi A, Allahverdi Pourfallah T, Shahani S. Radioprotective effect of chicory seeds against genotoxicity induced by ionizing radiation in human normal lymphocytes. Cell Mol Biol (Noisy-le-grand). 2015;61(4):46-50.

84. Hou J, Wang F, Kong P, Yu PK, Wang H, Han W. Gene profiling characteristics of radioadaptive response in AG01522 normal human fibroblasts. PLoS One. 2015;10(4):e0123316.

85. Litvinchuk AV, Vachelova J, Michaelidesova A, Wagner R, Davidkova M. Dose-dependent micronuclei formation in normal human fibroblasts exposed to proton radiation. Radiat Environ Biophys. 2015;54(3):327-34.

86. Lusiyanti Y, Alatas, Z., Syaifudin, M. Lack of Radioprotective Potential of Ginseng in Suppressing Micronuclei Frequency in Human Blood Lymphocyte under Gamma Irradiation. HAYATI Journal of Biosciences 2015;22(2):93-7.

87. Miszczyk J, Rawojc K, Panek A, Swakon J, Prasanna PG, Rydygier M. Response of human lymphocytes to proton radiation of 60 MeV compared to 250 kV X-rays by the cytokinesis-block micronucleus assay. Radiother Oncol. 2015;115(1):128-34.

88. Pajic J, Rakic B, Rovcanin B, Jovicic D, Novakovic I, Milovanovic A, et al. Inter-individual variability in the response of human peripheral blood lymphocytes to ionizing radiation: comparison of the dicentric and micronucleus assays. Radiat Environ Biophys. 2015;54(3):317-25.

89. Shahani S, Rostamnezhad M, Ghaffari-Rad V, Ghasemi A, Allahverdi Pourfallah T, Hosseinimehr SJ. Radioprotective Effect of Achillea millefolium L Against Genotoxicity Induced by Ionizing Radiation in Human Normal Lymphocytes. Dose Response. 2015;13(1):1559325815583761.

90. Shirani S, Mozdarani, H., Mahmoodzadeh, A., Salimi, M. Radio-adaptive response of peripheral blood lymphocytes following bystander effects induced by preirradiated CHO-K1 cells using the micronucleus assay. Int J Radiat Res. 2015;13(2):151-6.

91. Snijders AM, Mannion BJ, Leung SG, Moon SC, Kronenberg A, Wiese C. Micronucleus formation in human keratinocytes is dependent on radiation quality and tissue architecture. Environ Mol Mutagen. 2015;56(1):22-31.

92. Tamizh Selvan G, Chaudhury NK, Venkatachalam P. Comparison of results of the manual and automated scoring of micronucleus frequencies in (60)Co-irradiated peripheral blood lymphocytes for triage dosimetry. Appl Radiat Isot. 2015;97:70-7.

93. Widel M, Lalik A, Krzywon A, Poleszczuk J, Fujarewicz K, Rzeszowska-Wolny J. The different radiation response and radiation-induced bystander effects in colorectal carcinoma cells differing in p53 status. Mutat Res. 2015;778:61-70.

94. Alcaraz M, Alcaraz-Saura M, Achel DG, Olivares A, Lopez-Morata JA, Castillo J. Radiosensitizing effect of rosmarinic acid in metastatic melanoma B16F10 cells. Anticancer Res. 2014;34(4):1913-21.

95. Alcaraz M, Quesada S, Armero D, Martin-Gil R, Olivares A, Achel GD. Genotoxicity and cytotoxicity of sevoflurane in two human cell lines in vitro with ionizing radiation. Colomb Med (Cali). 2014;45(3):104-9.

96. Balajee AS, Bertucci A, Taveras M, Brenner DJ. Multicolour FISH analysis of ionising radiation induced micronucleus formation in human lymphocytes. Mutagenesis. 2014;29(6):447-55.

97. Brehwens K, Bajinskis A, Haghdoost S, Wojcik A. Micronucleus frequencies and clonogenic cell survival in TK6 cells exposed to changing dose rates under controlled temperature conditions. Int J Radiat Biol. 2014;90(3):241-7.

98. Chaurasia RK, Balakrishnan S, Kunwar A, Yadav U, Bhat N, Anjaria K, et al. Cyto-genotoxicity assessment of potential radioprotector, 3,3'-diselenodipropionic acid (DSePA) in Chinese Hamster Ovary (CHO) cells and human peripheral blood lymphocytes. Mutat Res Genet Toxicol Environ Mutagen. 2014;774:8-16.

99. Cinkilic N, Tuzun E, Cetintas SK, Vatan O, Yilmaz D, Cavas T, et al. Radio-protective effect of cinnamic acid, a phenolic phytochemical, on genomic instability induced by X-rays in human blood lymphocytes in vitro. Mutat Res Genet Toxicol Environ Mutagen. 2014;770:72-9.

100. Dutta S, Gupta ML. Alleviation of radiation-induced genomic damage in human peripheral blood lymphocytes by active principles of Podophyllum hexandrum: an in vitro study using chromosomal and CBMN assay. Mutagenesis. 2014;29(2):139-47.

101. Leskovac A, Petrovic S, Guc-Scekic M, Vujic D, Joksic G. Radiation-induced mitotic catastrophe in FANCD2 primary fibroblasts. Int J Radiat Biol. 2014;90(5):373-81.

102. Pei H, Chen W, Hu W, Zhu M, Liu T, Wang J, et al. GANRA-5 protects both cultured cells and mice from various radiation types by functioning as a free radical scavenger. Free Radic Res. 2014;48(6):670-8.

103. Ryu TH, Kim, J., Kim, J.K. Radiation exposure dose in human blood lymphocytes as assessed by the CBMN assay. Journal of Ecology and Environment. 2014;37:195-200.

104. Santos GS, Tsutsumi S, Vieira DP, Bartolini P, Okazaki K. Effect of Brazilian propolis (AF-08) on genotoxicity, cytotoxicity and clonogenic death of Chinese hamster ovary (CHO-K1) cells irradiated with (60)Co gamma-radiation. Mutat Res Genet Toxicol Environ Mutagen. 2014;762:17-23.

105. Selvan GT, Bhavani M, Vijayalakshmi J, Paul Solomon FD, Chaudhury NK, Venkatachalam P. Delayed mitogenic stimulation decreases DNA damage assessed by micronucleus assay in human peripheral blood lymphocytes after (60)co irradiation. Dose Response. 2014;12(3):498-508.

106. Seth I, Schwartz JL, Stewart RD, Emery R, Joiner MC, Tucker JD. Neutron exposures in human cells: bystander effect and relative biological effectiveness. PLoS One. 2014;9(6):e98947.

107. Vandersickel V, Beukes P, Van Bockstaele B, Depuydt J, Vral A, Slabbert J. Induction and disappearance of gammaH2AX foci and formation of micronuclei after exposure of human lymphocytes to (6)(0)Co gamma-rays and p(66)+ Be(40) neutrons. Int J Radiat Biol. 2014;90(2):149-58.

108. Zhao H, Lu X, Li S, Chen DQ, Liu QJ. Characteristics of nucleoplasmic bridges induced by 60Co gamma-rays in human peripheral blood lymphocytes. Mutagenesis. 2014;29(1):49-51.

109. Cheong HS, Seth I, Joiner MC, Tucker JD. Relationships among micronuclei, nucleoplasmic bridges and nuclear buds within individual cells in the cytokinesis-block micronucleus assay. Mutagenesis. 2013;28(4):433-40.

110. Ren R, He M, Dong C, Xie Y, Ye S, Yuan D, et al. Dose response of micronuclei induced by combination radiation of alpha-particles and gamma-rays in human lymphoblast cells. Mutat Res. 2013;741-742:51-6.

111. Acharya S, Narayana, Y., Joseph, P., Sanjeev, G., Bhat, N.N. Assessment of cell damages induced by gamma rays and pulsed electron beam at different dose rates. CARS2011: conference on accelerator radiation safety; Mumbai (India); 16-18 Nov 2011. 2011;117:103.

112. Begum N, Prasad NR, Kanimozhi G, Hasan AQ. Apigenin ameliorates gamma radiation-induced cytogenetic alterations in cultured human blood lymphocytes. Mutat Res. 2012;747(1):71-6.

113. Heshmati E, Mozdarani, H., Abdolmaleki, P., Khoshaman, K. Radiosensitizing effects of gemcitabine on aerobic and chronically hypoxic HeLa and MRC5 cells in-vitro. Int J Radiat Res. 2012;10(1):11-8.

114. Schmid TE, Greubel C, Hable V, Zlobinskaya O, Michalski D, Girst S, et al. Low LET protons focused to submicrometer shows enhanced radiobiological effectiveness. Phys Med Biol. 2012;57(19):5889-907.

115. Staaf E, Brehwens K, Haghdoost S, Nievaart S, Pachnerova-Brabcova K, Czub J, et al. Micronuclei in human peripheral blood lymphocytes exposed to mixed beams of X-rays and alpha particles. Radiat Environ Biophys. 2012;51(3):283-93.

116. Zuo YH, Dang XH, Zhang HF, Liu JG, Duan ZK, Wang ZW, et al. Genomic instability induced by ionizing radiation in human hepatocytes. J Toxicol Environ Health A. 2012;75(12):700-6.

117. Aypar U, Morgan WF, Baulch JE. Radiation-induced epigenetic alterations after low and high LET irradiations. Mutat Res. 2011;707(1-2):24-33.

118. Belloni P, Latini P, Palitti F. Radiation-induced bystander effect in healthy G(o) human lymphocytes: biological and clinical significance. Mutat Res. 2011;713(1-2):32-8.

119. Hosseinimehr SJ, Mahmoudzadeh A, Ahmadi A, Ashrafi SA, Shafaghati N, Hedayati N. The radioprotective effect of Zataria multiflora against genotoxicity induced by gamma irradiation in human blood lymphocytes. Cancer Biother Radiopharm. 2011;26(3):325-9.

120. Konopacka M, Rogolinski J, Slosarek K. Direct and bystander effects induced by scattered radiation generated during penetration of radiation inside a water-phantom. Mutat Res. 2011;721(1):6-14.

121. Kalpana KB, Devipriya N, Thayalan K, Menon VP. Protection against X-ray radiation-induced cellular damage of human peripheral blood lymphocytes by an aminothiazole derivative of dendrodoine. Chem Biol Interact. 2010;186(3):267-74.

122. Schmid TE, Dollinger G, Hable V, Greubel C, Zlobinskaya O, Michalski D, et al. Relative biological effectiveness of pulsed and continuous 20 MeV protons for micronucleus induction in 3D human reconstructed skin tissue. Radiother Oncol. 2010;95(1):66-72.

123. Vandersickel V, Mancini M, Slabbert J, Marras E, Thierens H, Perletti G, et al. The radiosensitizing effect of Ku70/80 knockdown in MCF10A cells irradiated with X-rays and p(66)+Be(40) neutrons. Radiat Oncol. 2010;5:30.

124. Vandersickel V, Mancini M, Marras E, Willems P, Slabbert J, Philippe J, et al. Lentivirus-mediated RNA interference of Ku70 to enhance radiosensitivity of human mammary epithelial cells. Int J Radiat Biol. 2010;86(2):114-24.

125. Acharya S, Sanjeev G, Bhat NN, Siddappa K, Narayana Y. The effect of electron and gamma irradiation on the induction of micronuclei in cytokinesis-blocked human blood lymphocytes. Radiat Environ Biophys. 2009;48(2):197-203.

126. Du C, Gao Z, Venkatesha VA, Kalen AL, Chaudhuri L, Spitz DR, et al. Mitochondrial ROS and radiation induced transformation in mouse embryonic fibroblasts. Cancer Biol Ther. 2009;8(20):1962-71.

127. Yue J, Wang Q, Lu H, Brenneman M, Fan F, Shen Z. The cytoskeleton protein filamin-A is required for an efficient recombinational DNA double strand break repair. Cancer Res. 2009;69(20):7978-85.

128. Kalpana KB, Devipriya N, Srinivasan M, Menon VP. Investigation of the radioprotective efficacy of hesperidin against gamma-radiation induced cellular damage in cultured human peripheral blood lymphocytes. Mutat Res. 2009;676(1-2):54-61.

129. Ryabokon NI, Nikitchenko NV, Dalivelya OV, Goncharova RI, Duburs G, Konopacka M, et al. Modulation of cellular defense processes in human lymphocytes in vitro by a 1,4-dihydropyridine derivative. Mutat Res. 2009;679(1-2):33-8.

130. Singh S, Bala M, Kumar R, Kumar A, Dhiman SC. Modification in the expression of Mre11/Rad50/Nbs1 complex in low dose irradiated human lymphocytes. Dose Response. 2009;7(3):193-207.

131. Devipriya N, Sudheer AR, Srinivasan M, Menon VP. Quercetin ameliorates gamma radiation-induced DNA damage and biochemical changes in human peripheral blood lymphocytes. Mutat Res. 2008;654(1):1-7.

132. Kim KC, Jun HJ, Kim JS, Kim IG. Enhancement of radiation response with combined Ganoderma lucidum and Duchesnea chrysantha extracts in human leukemia HL-60 cells. Int J Mol Med. 2008;21(4):489-98.

133. Wojewodzka M, Iwanenko, T., Kruszewski, M., Machaj, E.K., Pojda, Z., Gozdzik, A., Oldak, T. DNA damage in subpopulations of human lymphocytes irradiated with doses in the range of 0-1 Gy of X-radiation. Nukleonika. 2008;53(4):145-9.

134. Zhang Y, Rohde LH, Emami K, Hammond D, Casey R, Mehta SK, et al. Suppressed expression of non-DSB repair genes inhibits gamma-radiation-induced cytogenetic repair and cell cycle arrest. DNA Repair (Amst). 2008;7(11):1835-45.

135. Gangopadhyay S, Karmakar P, Dasgupta U, Chakraborty A. Trifluoperazine stimulates ionizing radiation induced cell killing through inhibition of DNA repair. Mutat Res. 2007;633(2):117-25.

136. Groesser T, Chun E, Rydberg B. Relative biological effectiveness of high-energy iron ions for micronucleus formation at low doses. Radiat Res. 2007;168(6):675-82.

137. Kim CS, Kim JM, Nam SY, Yang KH, Jeong M, Kim HS, et al. Low-dose of ionizing radiation enhances cell proliferation via transient ERK1/2 and p38 activation in normal human lung fibroblasts. J Radiat Res. 2007;48(5):407-15.

138. Mozdarani H, Taheri, A., Haeri, S.A. Assessment of the radioprotective effects of amifostine on human lymphocytes irradiated in vitro by gamma-rays using cytokinesis-blocked micronucleus assay. Int J Radiat Res 2007. 2007;5(1):9-16.

139. Pathak R, Dey SK, Sarma A, Khuda-Bukhsh AR. Cell killing, nuclear damage and apoptosis in Chinese hamster V79 cells after irradiation with heavy-ion beams of (16)O, (12)C and (7)Li. Mutat Res. 2007;632(1-2):58-68.

140. Pathak R, Dey SK, Sarma A, Khuda-Bukhsh AR. Genotoxic effects in M5 cells and Chinese hamster V79 cells after exposure to 7Li-beam (LET=60 keV/microm) and correlation of their survival dynamics to nuclear damages and cell death. Mutat Res. 2007;628(1):56-66.

141. Del Bano MJ, Castillo J, Benavente-Garcia O, Lorente J, Martin-Gil R, Acevedo C, et al. Radioprotective-antimutagenic effects of rosemary phenolics against chromosomal damage induced in human lymphocytes by gamma-rays. J Agric Food Chem. 2006;54(6):2064-8.

142. Hori M, Kojima, S. Effect of 5,6,7,8-Tetrahydrobiopterin (BH4) on γ-Ray-induced Micronucleus Frequency in RAW264.7 Cells. Pteridines. 2013;17(3):74-81.

143. Lehnert A, Lessmann E, Pawelke J, Dorr W. RBE of 25 kV X-rays for the survival and induction of micronuclei in the human mammary epithelial cell line MCF-12A. Radiat Environ Biophys. 2006;45(4):253-60.

144. Prasad NR, Srinivasan M, Pugalendi KV, Menon VP. Protective effect of ferulic acid on gamma-radiation-induced micronuclei, dicentric aberration and lipid peroxidation in human lymphocytes. Mutat Res. 2006;603(2):129-34.

145. Srinivasan M, Rajendra Prasad N, Menon VP. Protective effect of curcumin on gamma-radiation induced DNA damage and lipid peroxidation in cultured human lymphocytes. Mutat Res. 2006;611(1-2):96-103.

146. Jagetia GC, Venkatesha VA. Effect of mangiferin on radiation-induced micronucleus formation in cultured human peripheral blood lymphocytes. Environ Mol Mutagen. 2005;46(1):12-21.

147. Rithidech KN, Tungjai M, Whorton EB. Protective effect of apigenin on radiation-induced chromosomal damage in human lymphocytes. Mutat Res. 2005;585(1-2):96-104.

148. Somodi Z, Zyuzikov NA, Kashino G, Trott KR, Prise KM. Radiation-induced genomic instability in repair deficient mutants of Chinese hamster cells. Int J Radiat Biol. 2005;81(12):929-36.

149. Yang H, Asaad N, Held KD. Medium-mediated intercellular communication is involved in bystander responses of X-ray-irradiated normal human fibroblasts. Oncogene. 2005;24(12):2096-103.

150. Akudugu JM, Theron T, Serafin AM, Bohm L. Influence of DNA double-strand break rejoining on clonogenic survival and micronucleus yield in human cell lines. Int J Radiat Biol. 2004;80(2):93-104.

151. Konopacka M, Rogolinski J. Thiamine prevents X-ray induction of genetic changes in human lymphocytes in vitro. Acta Biochim Pol. 2004;51(3):839-43.

152. Lee TK, Allison RR, O'Brien KF, Khazanie PG, Johnke RM, Brown R, et al. Ginseng reduces the micronuclei yield in lymphocytes after irradiation. Mutat Res. 2004;557(1):75-84.

153. Slowinski J, Bierzynska-Macyszyn, G., Mazurek, U., Widel, M., Latocha, M., Stomal, M., Snietura, M., Mrowka, R. Cytokinesis-block micronucleus assay in human glioma cells exposed to radiation. Image Analysis & Stereology. 2004;23(3).

154. Bhat NN, Rao BS. Dose rate effect on micronuclei induction in cytokinesis blocked human peripheral blood lymphocytes. Radiat Prot Dosimetry. 2003;106(1):45-52.

155. Jagetia GC, Venkatesh P, Baliga MS. Evaluation of the radioprotective effect of Aegle marmelos (L.) Correa in cultured human peripheral blood lymphocytes exposed to different doses of gamma-radiation: a micronucleus study. Mutagenesis. 2003;18(4):387-93.

156. Jagetia GC, Aruna R. Correlation of micronuclei-induction with the cell survival in HeLa cells treated with a base analogue, azidothymidine (AZT) before exposure to different doses of gamma-radiation. Toxicol Lett. 2003;139(1):33-43.

157. Slonina D, Spekl K, Panteleeva A, Brankovic K, Hoinkis C, Dorr W. Induction of micronuclei in human fibroblasts and keratinocytes by 25 kV x-rays. Radiat Environ Biophys. 2003;42(1):55-61.

158. Thomas P, Umegaki K, Fenech M. Nucleoplasmic bridges are a sensitive measure of chromosome rearrangement in the cytokinesis-block micronucleus assay. Mutagenesis. 2003;18(2):187-94.

159. Jagetia GC, Shrinath Baliga M. Vincristine increases the genomic instability in irradiated cultured human peripheral blood lymphocytes. Toxicol Lett. 2002;126(3):179-86.

160. Muller WU, Rode A. The micronucleus assay in human lymphocytes after high radiation doses (5-15 Gy). Mutat Res. 2002;502(1-2):47-51.

161. Schafer J, Bachtler J, Engling A, Little JB, Weber KJ, Wenz F. Suppression of apoptosis and clonogenic survival in irradiated human lymphoblasts with different TP53 status. Radiat Res. 2002;158(6):699-706.

162. Eastham AM, Atkinson J, West CM. Relationships between clonogenic cell survival, DNA damage and chromosomal radiosensitivity in nine human cervix carcinoma cell lines. Int J Radiat Biol. 2001;77(3):295-302.

163. Ponsa I, Barquinero JF, Miro R, Egozcue J, Genesca A. Non-disjunction and chromosome loss in gamma-irradiated human lymphocytes: a fluorescence in situ hybridization analysis using centromere-specific probes. Radiat Res. 2001;155(3):424-31.

164. Sgura A, Antoccia A, Cherubini R, Tanzarella C. Chromosome nondisjunction and loss induced by protons and X rays in primary human fibroblasts: role of centromeres in aneuploidy. Radiat Res. 2001;156(3):225-31.

165. Shao C, Aoki M, Furusawa Y. Medium-mediated bystander effects on HSG cells co-cultivated with cells irradiated by X-rays or a 290 MeV/u carbon beam. J Radiat Res. 2001;42(3):305-16.

166. Vral A, Thierens H, Bryant P, De Ridder L. A higher micronucleus yield in B-versus T-cells after low-dose gamma-irradiation is not linked with defective Ku86 protein. Int J Radiat Biol. 2001;77(3):329-39.

167. Yoshida K, Yamazaki H, Ozeki S, Inoue T, Yoshioka Y, Yoneda M, et al. Mitochondrial genotypes and radiation-induced micronucleus formation in human osteosarcoma cells in vitro. Oncol Rep. 2001;8(3):615-9.

168. Bishay K, Ory K, Lebeau J, Levalois C, Olivier MF, Chevillard S. DNA damage-related gene expression as biomarkers to assess cellular response after gamma irradiation of a human lymphoblastoid cell line. Oncogene. 2000;19(7):916-23.

169. He JL, Chen WL, Jin LF, Jin HY. Comparative evaluation of the in vitro micronucleus test and the comet assay for the detection of genotoxic effects of X-ray radiation. Mutat Res. 2000;469(2):223-31.

170. Jagetia GC, Adiga SK. Correlation between cell survival and micronuclei formation in V79 cells treated with vindesine before exposure to different doses of gamma-radiation. Mutat Res. 2000;448(1):57-68.

171. Joksic G, Pajovic SB, Stankovic M, Pejic S, Kasapovic J, Cuttone G, et al. Chromosome aberrations, micronuclei, and activity of superoxide dismutases in human lymphocytes after irradiation in vitro. Cell Mol Life Sci. 2000;57(5):842-50.

172. Adiga SK, Jagetia GC. Correlation between cell survival, micronuclei-induction, and LDH activity in V79 cells treated with teniposide (VM-26) before exposure to different doses of gamma radiation. Toxicol Lett. 1999;109(1-2):31-41.

173. Kriehuber R, Simko M, Schiffmann D, Trott KR. Delayed cytotoxic and genotoxic effects in a human cell line following X-irradiation. Int J Radiat Biol. 1999;75(8):1021-7.

174. Mariya Y, Abe, Y., Streffer, C. Utility of the cytokinesis-block micronucleus assay to assess combined effects of irradiation and cisplatin. Hirosaki Igaku. 1999;50(4):215-22.

175. Guo GZ, Sasai K, Oya N, Takagi T, Shibuya K, Hiraoka M. Simultaneous evaluation of radiation-induced apoptosis and micronuclei in five cell lines. Int J Radiat Biol. 1998;73(3):297-302.

176. Keshava C, Keshava N, Ong TM, Nath J. Protective effect of vanillin on radiation-induced micronuclei and chromosomal aberrations in V79 cells. Mutat Res. 1998;397(2):149-59.

177. Takagi T, Sasai K, Shibamoto Y, Akagi K, Oya N, Shibata T, et al. The influence of DNA ploidy of a human tumor cell line on the frequencies of micronuclei or chromosome aberrations after irradiation. Mutat Res. 1998;418(1):49-57.

178. Vral A, Louagie H, Thierens H, Philippe J, Cornelissen M, de Ridder L. Micronucleus frequencies in cytokinesis-blocked human B lymphocytes after low dose gamma-irradiation. Int J Radiat Biol. 1998;73(5):549-55.

179. Wuttke K, Muller WU, Streffer C. The sensitivity of the in vitro cytokinesis-blocked micronucleus assay in lymphocytes for different and combined radiation qualities. Strahlenther Onkol. 1998;174(5):262-8.

180. Catena C, Asprea L, Carta S, Tortora G, Conti D, Parasacchi P, et al. Dose-response of X-irradiated human and equine lymphocytes. Mutat Res. 1997;373(1):9-16.

181. Kim SH, Han DU, Lim JT, Jo SK, Kim TH. Induction of micronuclei in human, goat, rabbit peripheral blood lymphocytes and mouse splenic lymphocytes irradiated in vitro with gamma radiation. Mutat Res. 1997;393(3):207-14.

182. Manti L, Jamali M, Prise KM, Michael BD, Trott KR. Genomic instability in Chinese hamster cells after exposure to X rays or alpha particles of different mean linear energy transfer. Radiat Res. 1997;147(1):22-8.

183. Paul SF, Venkatachalam P, Jeevanram RK. A comparative study of synchronised and conventional culture methods on the micronucleus dose-response curve. Mutat Res. 1997;391(1-2):91-8.

184. Paul SF, Venkatachalam P, Jeevanram RK. Analysis of radiation dose-response curve obtained with cytokinesis block micronucleus assay. Nucl Med Biol. 1997;24(5):413-6.

185. Vral A, Thierens H, De Ridder L. In vitro micronucleus-centromere assay to detect radiation-damage induced by low doses in human lymphocytes. Int J Radiat Biol. 1997;71(1):61-8.

186. Darroudi F, Meijers CM, Hadjidekova V, Natarajan AT. Detection of aneugenic and clastogenic potential of X-rays, directly and indirectly acting chemicals in human hepatoma (Hep G2) and peripheral blood lymphocytes, using the micronucleus assay and fluorescent in situ hybridization with a DNA centromeric probe. Mutagenesis. 1996;11(5):425-33.

187. Gajdusek CM, Tian H, London S, Zhou D, Rasey J, Mayberg MR. Gamma radiation effect on vascular smooth muscle cells in culture. Int J Radiat Oncol Biol Phys. 1996;36(4):821-8.

188. Gaziev AI, Sologub GR, Fomenko LA, Zaichkina SI, Kosyakova NI, Bradbury RJ. Effect of vitamin-antioxidant micronutrients on the frequency of spontaneous and in vitro gamma-ray-induced micronuclei in lymphocytes of donors: the age factor. Carcinogenesis. 1996;17(3):493-9.

189. Keshava C, Nagalakshmi R, Ong T, Nath J. Inhibitory effect of folinic acid on radiation-induced micronuclei and chromosomal aberrations in V79 cells. Mutat Res. 1996;352(1-2):123-34.

190. Koksal G, Dalci DO, Pala FS. Micronuclei in human lymphocytes: the Co-60 gamma-ray dose-response. Mutat Res. 1996;359(2):151-7.

191. Mill AJ, Wells J, Hall SC, Butler A. Micronucleus induction in human lymphocytes: comparative effects of X rays, alpha particles, beta particles and neutrons and implications for biological dosimetry. Radiat Res. 1996;145(5):575-85.

192. Vijayalaxmi, Reiter RJ, Herman TS, Meltz ML. Melatonin and radioprotection from genetic damage: in vivo/in vitro studies with human volunteers. Mutat Res. 1996;371(3-4):221-8.

193. Courdi A, Mari D, Herault J, Chauvel P. Micronucleus induction and reproductive death in a human cell line exposed to low-energy argon beam. Radiat Environ Biophys. 1995;34(2):85-9.

194. Slavotinek A, Miller E, Taylor GM, Nusse M, van Heyningen V. Micronucleus frequencies in lymphoblastoid cell lines measured with the cytokinesis-block technique and flow cytometry. Mutagenesis. 1995;10(5):439-45.

195. Vijayalaxmi, Leal BZ, Deahl TS, Meltz ML. Variability in adaptive response to low dose radiation in human blood lymphocytes: consistent results from chromosome aberrations and micronuclei. Mutat Res. 1995;348(1):45-50.

196. Catena C, Conti D, Villani P, Nastasi R, Archilei R, Righi E. Micronuclei and 3AB index in human and canine lymphocytes after in vitro X-irradiation. Mutat Res. 1994;312(1):1-8.

197. Ono K, Masunaga S, Akaboshi M, Akuta K. Estimation of the initial slope of the cell survival curve after irradiation from micronucleus frequency in cytokinesis-blocked cells. Radiat Res. 1994;138(1 Suppl):S101-4.

198. Silva MJ, Carothers A, Dias A, Luis JH, Piper J, Boavida MG. Dose dependence of radiation-induced micronuclei in cytokinesis-blocked human lymphocytes. Mutat Res. 1994;322(2):117-28.

199. Verhaegen F, Vral A. Sensitivity of micronucleus induction in human lymphocytes to low-LET radiation qualities: RBE and correlation of RBE and LET. Radiat Res. 1994;139(2):208-13.

200. Bush C, McMillan TJ. Micronucleus formation in human tumour cells: lack of correlation with radiosensitivity. Br J Cancer. 1993;67(1):102-6.

201. Hurwitz SJ, Hlatky L. Assessment of radiation response on a cell-by-cell basis using in situ densitometric imaging of micronuclei. Radiat Res. 1993;134(1):112-6.

202. Littlefield LG, Joiner EE, Colyer SP, Sallam F, Frome EL. Concentration-dependent protection against X-ray-induced chromosome aberrations in human lymphocytes by the aminothiol WR-1065. Radiat Res. 1993;133(1):88-93.

203. Slavotinek A, McMillan TJ, Steel CM. A comparison of micronucleus frequency and radiation survival in lymphoblastoid cell lines. Mutagenesis. 1993;8(6):569-75.

204. Darroudi F, Farooqi Z, Benova D, Natarajan AT. The mouse splenocyte assay, an in vivo/in vitro system for biological monitoring: studies with X-rays, fission neutrons and bleomycin. Mutat Res. 1992;272(3):237-48.

205. Armitage MP, Bryant PE, Riches AC. Cytogenetic responses of human uroepithelial cell lines and a malignant bladder carcinoma cell line to X-rays. Mutagenesis. 1991;6(6):515-8.

206. Balasem AN, Ali AS. Establishment of dose-response relationships between doses of Cs-137 gamma-rays and frequencies of micronuclei in human peripheral blood lymphocytes. Mutat Res. 1991;259(2):133-8.

207. Gantenberg HW, Wuttke K, Streffer C, Muller WU. Micronuclei in human lymphocytes irradiated in vitro or in vivo. Radiat Res. 1991;128(3):276-81.

208. Odagiri Y, Dempsey JL, Morley AA. Damage to lymphocytes by X-ray and bleomycin measured with the cytokinesis-block micronucleus technique. Mutat Res. 1990;237(3-4):147-52.

209. Erexson GL, Kligerman AD, Halperin EC, Honore GM, Allen JW. Micronuclei in binucleated lymphocytes of mice following exposure to gamma radiation. Environ Mol Mutagen. 1989;13(2):128-32.

210. Köksal G, Lloyd, D.C., Edwards, A.A., Prosser, J.S. The Dependence the Micronucleus Yield in Human Lymphocytes on Culture and Cytokinesis Blocking Times. Radiation Protection Dosimetry. 1989;29(3):209-12.

211. Kormos C, Koteles GJ. Micronuclei in X-irradiated human lymphocytes. Mutat Res. 1988;199(1):31-5.

212. Prosser JS, Moquet JE, Lloyd DC, Edwards AA. Radiation induction of micronuclei in human lymphocytes. Mutat Res. 1988;199(1):37-45.

213. Ramalho A, Sunjevaric I, Natarajan AT. Use of the frequencies of micronuclei as quantitative indicators of X-ray-induced chromosomal aberrations in human peripheral blood lymphocytes: comparison of two methods. Mutat Res. 1988;207(3-4):141-6.

214. Go YJ, Shin, K.S., Jeong, K.S., Park, S.J., Kim, S.H., Ryu, S.Y., Kim, C.H., Kim, E.J., Kang, C.M., Kim, T.H. Dose estimation with the calibration of dose-response curve of micronucleus in human peripheral lymphocytes induced by 50MeV proton beams. Int J Radiat Res 2011. 2011;8(4):231-6.

215. Buglewicz DJ, Banks AB, Hirakawa H, Fujimori A, Kato TA. Monoenergetic 290 MeV/n carbon-ion beam biological lethal dose distribution surrounding the Bragg peak. Sci Rep. 2019;9(1):6157.

216. Shao C, Furusawa Y, Matsumoto Y, Pan Y, Xu P, Chen H. Effect of gap junctional intercellular communication on radiation responses in neoplastic human cells. Radiat Res. 2007;167(3):283-8.

217. Kalanxhi E, Dahle J. The role of serotonin and p53 status in the radiation-induced bystander effect. Int J Radiat Biol. 2012;88(10):773-6.

218. Shao C, Folkard M, Held KD, Prise KM. Estrogen enhanced cell-cell signalling in breast cancer cells exposed to targeted irradiation. BMC Cancer. 2008;8:184.

219. Nelson JM, Brooks AL, Metting NF, Khan MA, Buschbom RL, Duncan A, et al. Clastogenic effects of defined numbers of 3.2 MeV alpha particles on individual CHO-K1 cells. Radiat Res. 1996;145(5):568-74.
